# Exchange Bias in a Dinuclear Erbium Single-Molecule Magnet Bridged by a Helicene Ligand

**DOI:** 10.1021/acs.inorgchem.5c01992

**Published:** 2025-07-17

**Authors:** Gabriela Handzlik, Mikołaj Żychowicz, Katarzyna Rzepka, Dawid Pinkowicz

**Affiliations:** † Faculty of Chemistry, 37799 Jagiellonian University, Gronostajowa 2, 30-387 Kraków, Poland

## Abstract

We report the synthesis and magneto-structural characterization of a dimeric Er-based single-molecule magnet (**Er**_**2**_) containing two [6]­helicene-1,2-diol dianions as bridging ligands. Diamagnetic dilution with yttrium cations at the synthesis stage leads to a composition that can be approximated as **ErY@Y**_**2**_. This enables a direct comparison of the slow magnetic relaxation of these closely related single-molecule magnets (SMMs): dimeric **Er**_**2**_ with erbium–erbium magnetic interactions and **ErY@Y**_**2**_ where these interactions are absent and yet the coordination sphere of the lanthanide remains identical to that in the dimer. The antiferromagnetic Er–Er interactions in **Er**_**2**_ result in exchange bias accelerating the magnetic relaxation around the crossing magnetic field, while their absence in **ErY@Y**_**2**_ enables longer relaxation times and leads to a less complex magnetic behavior.

## Graphical abstract

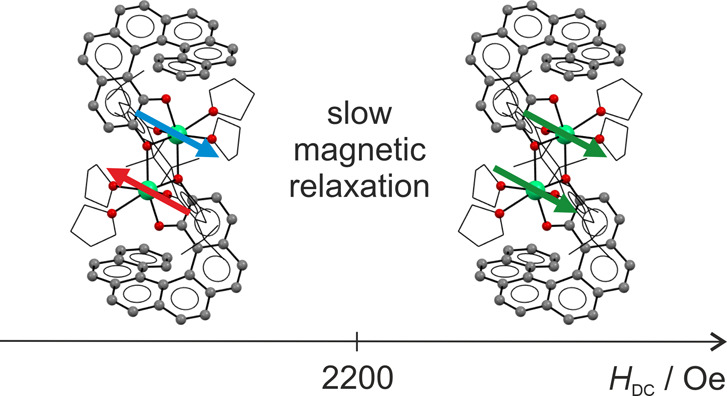

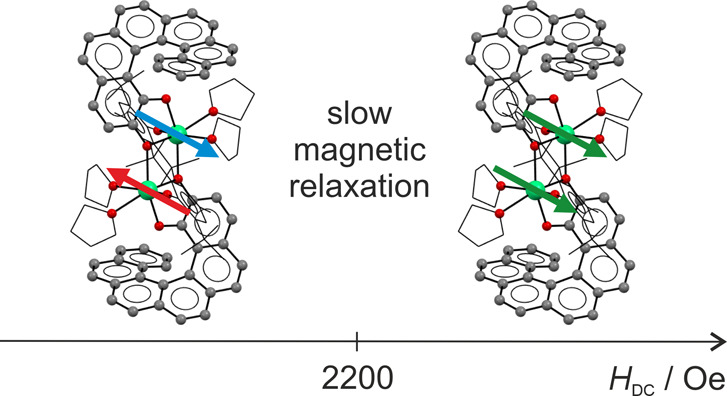

## Introduction

Single-molecule magnets (SMMs) are magnetic objects showing magnetic memory at a molecular level. In other words, a SMM behaves like a single magnetic domain, but has a perfectly defined structure and its size is of only several angstroms. The first generation of SMMs^1–9^ was designed as multinuclear molecules based on transition metal ions. The intention was to achieve a high total spin and magnetic moment through strong coupling of *d*-block metal ions, which would directly enhance the energy barrier for spin reversal and the blocking temperature (magnetic memory temperature).^10,11^ An alternative way to increase this energy barrier is through increasing the magnetic anisotropy of the system. However, it is very challenging to control magnetic anisotropy in multinuclear clusters or molecules.^12,13^ Hence, since the discovery of TbPc_2_ in 2003,^14^ the research efforts focused on single lanthanide ions for the construction of SMMs as they combine an easily tunable strong magnetic anisotropy and large magnetic moments. The design and construction of multinuclear lanthanide SMMs (Ln-SMMs) is hindered due to much weaker exchange interactions between lanthanide ions caused by the shielding of the 4*f* valence orbitals by the 5*p* and 5*s* orbitals.^15^ Typical exchange coupling constants *J* for polymetallic Ln-based complexes with diamagnetic bridges are usually smaller than 1 cm^–1^,^16^ leading to slow magnetic relaxation driven by single-ion effects^17^ with an interesting exception of single-molecule toroics, a special case of polynuclear lanthanide-based nanomagnets, where suitable magnetic interactions are required to stabilize the toroidal arrangement of magnetic moments of Ln ions.^18^ Nevertheless, significant research efforts in the field of Ln-SMMs are still almost exclusively focused on modeling the crystal field of individual lanthanide ions to maximize their intrinsic magnetic anisotropy.^19^

Lanthanide complexes with very short bridges between 4*f* metal ions provide an opportunity to study the magnetic interactions between these ions, with dinuclear complexes being particularly convenient and popular model systems.^20–22^ Coupling of the lanthanide ions can result in even higher total magnetic moments as compared to single ions and can minimize quantum tunneling of magnetization (QTM) at low temperatures.^23,24^ However, designing multinuclear SMMs is synthetically challenging because the ligand field for each metal center within such a dimer needs to generate a high magnetic anisotropy for that particular ion, and at the same time the orientation of the anisotropy axes of each individual ion must be carefully controlled with respect to each other e.g. parallel^25^ or toroidal.^18^ Noteworthy, in addition to multinuclear lanthanide-based SMMs bridged by diamagnetic molecular bridges, this family is also enriched by molecules bridged or coordinated by radicals.^21,24,26–38^ This type of bridging allows for strong ferromagnetic coupling of the lanthanide magnetic moments and in some cases can lead to large magnetic hysteresis with record coercive field values.^24,36^

Furthermore, the choice of ligands in the design of SMMs offers the opportunity to incorporate additional physical properties (e.g., chirality) into the molecule.^39^ Helicenes, as inherently axially chiral polycyclic molecules with high specific rotation values, are unique building blocks that can introduce chirality into the metal complex.^40,41^ The use of helicenes as ligands can allow the introduction of additional functionality to the compound in the form of physical effects arising from the chirality itself (e.g., natural circular dichroism or circularly polarized luminescence). In certain cases, it can even lead to the creation of second-order effects arising from the combination of chirality and magnetism (e.g., magneto-chiral dichroism).^42–44^ However, the field of lanthanide-based complexes with helicenes has only recently begun to be explored.^41–51^

Herein, we report the synthesis of the [Er^III^(hel)(BHT)(THF)_2_]_2_·2THF dimer (**Er**_**2**_; H_2_hel = [6]­helicene-1,2-diol; BHT = butylated hydroxytoluene) where the two Er^III^ cations are bridged by helicene ligands. Our goal in using helicenes in the design of metal ion complexes is to introduce strong chirality into the molecular materials through these unique building blocks. Herein, the racemic mixture of helicene was used as the first step. **Er**_**2**_ exhibits field-induced single-molecule magnet characteristics. Usually, to estimate the exchange interactions between lanthanide centers (*J*_Ln–Ln_) in dinuclear molecules, the analog with isotropic gadolinium ions is prepared and studied.^16,52–54^ However, it is also possible to explore magnetic interactions by preparing analogs highly diluted with a diamagnetic ion such as yttrium, lanthanum or lutetium.^55^ We chose to prepare the diamagnetic dilution by doping the yttrium-based analog with erbium ions during the synthesis. **ErY@Y**_**2**_ was prepared as a dilution of about 10% Er^III^ ions in the diamagnetic Y^III^ analog, which should result in a significant dominance of the ErY form (18%) over the Er_2_ dimers (1%) due to the statistical occupancy of the metal centers, leading to a single-ion SMM type of behavior of **ErY@Y**_**2**_. Structural, magnetic and theoretical characterization of **Er**_**2**_ and **ErY@Y**_**2**_ provide the understanding of the slow magnetic relaxation of these complexes and the nature of the interactions between paramagnetic erbium ions in **Er**_**2**_. Diamagnetic dilution enables the analysis of what happens when there is only one paramagnetic center in the dimer and erbium–erbium interactions are effectively switched-off, so they do not affect the thermal relaxation of the magnetization. While magnetic studies for Er_2_ ^20,25,56–59^ and Dy_2_ ^23,52,55,60–66^ dimers with phenolate or alkoxide bridging units are quite common and reveal both ferromagnetic or antiferromagnetic coupling depending on the Ln–O–Ln angle^63,66^ and Ln ion type,^65^ the related catecholate-bridged compounds equipped with an integrated helicene backbone are missing.

## Results and Discussion

### Synthesis

The **Er**_**2**_ complex is formed in a redox-type reaction between an Er­(BHT)_3_ complex^67^ (BHT = butylated hydroxytoluene) and [6]­helicene *o*-quinone in THF at room temperature (Scheme 1). The reduction potential for the *o*-quinone to the semiquinone radical anion is *E*° = −1.00 V (vs Fc^+/0^) and further reduction to the catecholate dianion requires *E*° = −1.71 V (vs Fc^+/0^).^68^ BHT is known to be a common antioxidant^69^ added to many foods and cosmetics.^70^ Neutral BHT can reduce Ag^+^ to Ag^71^ or Cu^2+^ to Cu^+^ ^72^ (in both cases *E*° > −0.7 V vs Fc^+/0^). The sodium salt of BHT has even stronger reducing properties and acts as a hydride source.^73^ In our work, two BHT anions coordinated to the Er center in Er^III^(BHT)_3_ are used for the reduction of a [6]­helicene-1,2-dione molecule to its catecholate form and then leave the coordination sphere of the Er center in this reaction. The synthesized compounds were characterized by single crystal X-ray diffraction, powder X-ray diffraction, infrared spectroscopy and SQUID magnetometry. The experimental results were supported by theoretical calculations for a better understanding of the physical phenomena. All experimental details are described in the Supporting Information (SI).

**Scheme 1:**
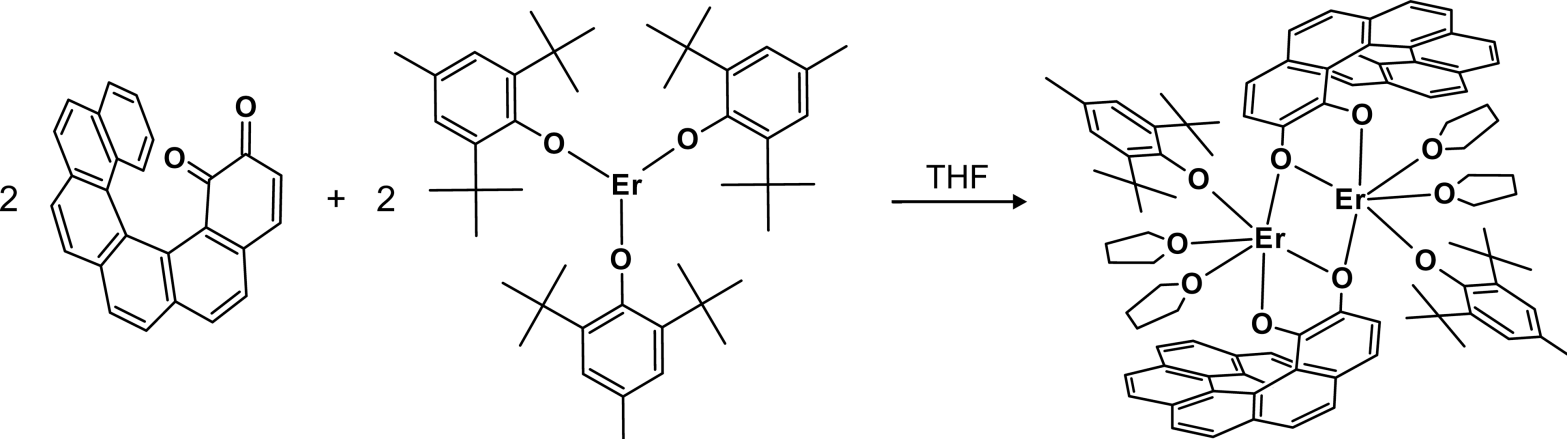
Reaction Scheme for the Synthesis of **Er**_**2**_ Complex

### Structural and Spectroscopic Characterization

The crystal structures of **Er**_**2**_ and **ErY@Y**_**2**_ were determined by single crystal X-ray diffraction at 100 K. Structural analysis for **Er**_**2**_ was also performed at 270 K for comparison with powder X-ray diffraction patterns of bulk **Er**_**2**_ and **ErY@Y**_**2**_ samples measured at room temperature to confirm the purity of the crystal phases. **Er**_**2**_ and **ErY@Y**_**2**_ are isostructural and crystallize in the centrosymmetric monoclinic space group *P*2_1_/*c*. The crystal structure solution and refinement parameters for **Er**_**2**_ (at 100 and 270 K) and **ErY@Y**_**2**_ (at 100 K) are presented in Table S1. The crystal structure of the dimer is discussed in detail below using **Er**_**2**_ at 100 K as an example.

The asymmetric unit consists of a half of the centrosymmetric Er-based dimer [Er^III^(hel)­(BHT)­(THF)_2_] and one crystallization THF molecule (Figure S1). In the dimer, each erbium ion (Er1) is coordinated by a total of six oxygen atoms and its coordination sphere has a significantly distorted octahedral geometry (according to the continuous shape measurement (CShM) analysis presented in Table S2).^74^ Three oxygen atoms coordinated to Er1 (O1, O2 and O1 from the second half of the dimer) come from two helicene-1,2-diol dianions and are almost in the same plane with the Er1 atom, forming a *mer* arrangement. The other three oxygen atoms coordinated to Er1 come from a BHT anion (O3) and two THF coordination molecules (O4 and O5). The comparison of the Er–O bond lengths is shown in Table S3. The distance between the erbium ion and the oxygen from the BHT ligand (Er1–O3) in the dimer is equal to 2.112(4) Å at 100 K, which is slightly longer than the Er–O distance in the Er­(BHT)_3_ precursor at 120 K (2.040(2)–2.048(3) Å)^67^ and quite similar to the alternative Er­(BHT)_3_(THF) precursor at 100 K (2.072(3)–2.098(5) Å).^38^

In the dimer, two Er ions (Er1) are bridged by two oxygen atoms (O1) from two helicene-1,2-diol dianions (Figure 1). The inversion center is in the center of the Er1–O1–Er1–O1 diamond, so each dimer contains both helicene enantiomers (*M* and *P*). The distance between two Er1 atoms is 3.7151(5) Å and the distance between two bridging oxygen O1 atoms is 2.636(6) Å. The Er1–O1 bond length measured between atoms from the same asymmetric unit is not significantly longer (2.283(5) Å) than the Er1–O1 bond length between atoms from two adjacent asymmetric units (2.273(5) Å). The O1–Er1–O1 and Er1–O1–Er1 angles in this bridging mode are 70.7(2)° and 109.3(2)°, respectively.

**Figure 1:**
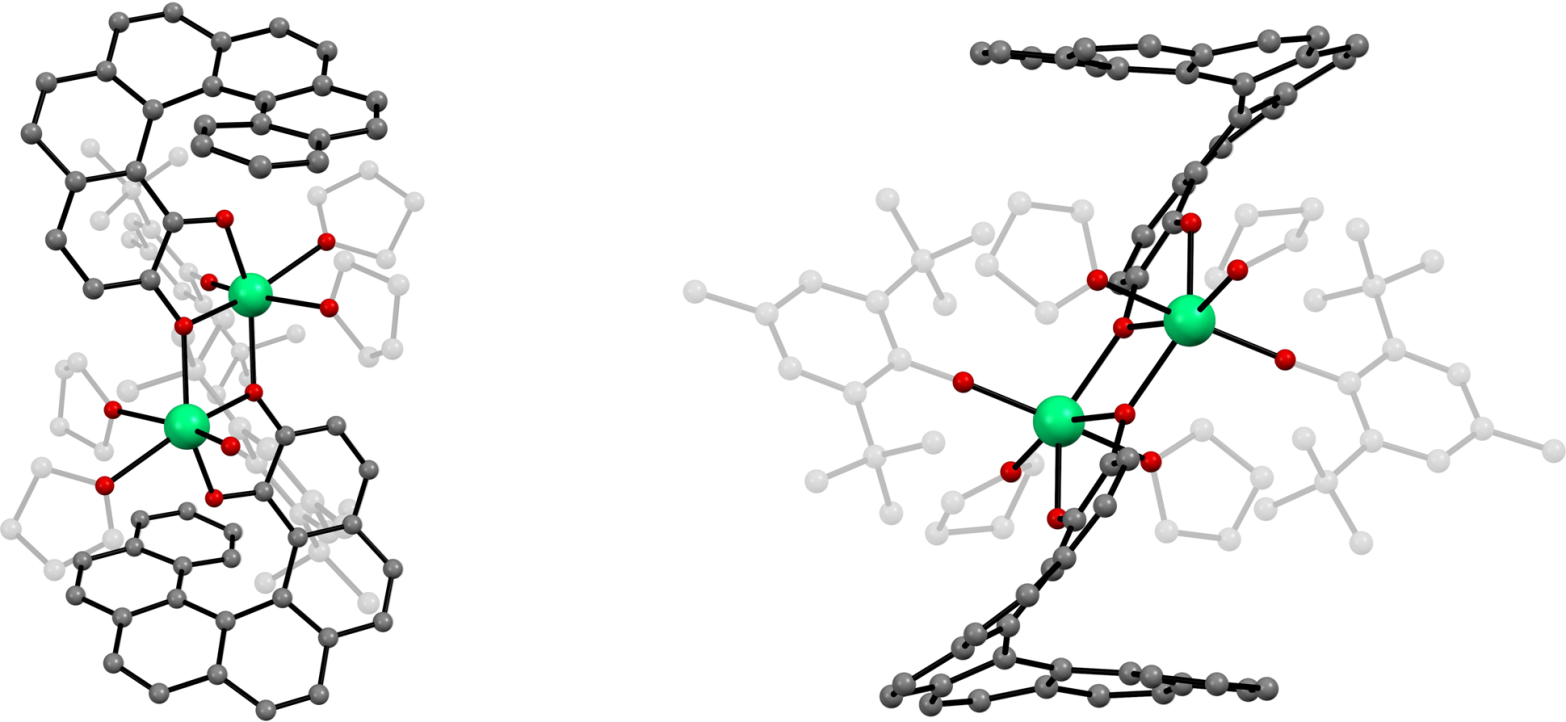
Crystal structure diagrams of the **Er**_**2**_ molecule viewed in two different orientations (Er–green, O–red, C–gray, H–omitted; parts of the molecule are dimmed for clarity).

The unit cell (V = 4331.59 Å^3^) contains two Er-based dimer molecules and four crystallization THF molecules. The Er–Er axis in the dimer aligns almost perfectly with the *a* crystallographic axis. The shortest Er–Er distance between atoms from two different dimers is 9.8605(7) Å which is sufficient to assume negligible intermolecular magnetic interactions in **Er**_**2**_ and **ErY@Y**_**2**_. The crystal structure is stabilized by weak hydrogen interactions described in detail in SI (Figures S2–S5).

The bond lengths of the catecholate groups of the helicene ligand (1.375(9) and 1.322(9) Å, respectively) coordinated to the erbium ions in **Er**_**2**_ fully confirm that diketone was reduced to a catecholate form during the reaction with Er­(BHT)_3_. The C–O bond lengths for this particular helicene molecule in the free diol form are equal to 1.362(4) and 1.346(4) Å and those for the free diketone form are equal to 1.211(3) and 1.210(2) Å.^68^ The C–O bond lengths in similar catecholate-type ligands are within the 1.30–1.35 Å range, according to the CSD database (measurements at low temperature).^75–78^ The observed difference of approximately 0.05 Å in the catecholate C–O bond lengths within the **Er**_**2**_ molecule (1.375(9) Å vs 1.322(9) Å) is most likely attributed to structural strain within the central Er–O–Er–O diamond-shaped core and within the five-membered C–O–Er–O–C rings formed by the catecholate group and erbium ions.

The isostructural character of the bulk **Er**_**2**_ and **ErY@Y**_**2**_ samples was confirmed by powder X-ray diffraction (pXRD; Figure S6). PXRD experiments were performed at room temperature for ground samples loaded into narrow-diameter (0.5 mm) borosilicate-glass capillaries under small amount of mother solution. The reflections on the experimental pXRD diffraction patterns are in good agreement with the simulated one from the single crystal X-ray diffraction measurement for **Er**_**2**_ at 270 K, confirming the identity and crystal phase purity of the samples.

Infrared spectroscopy (IR) was employed to analyze the differences between the helicene-1,2-dione used in the synthesis and the helicene-type ligand built into the final complexes **Er**_**2**_ and **ErY@Y**_**2**_. It also proved the chemical identity of both complexes. IR spectra (Figure S7) were collected in the 4000–675 cm^–1^ range. Each sample was measured as a thin layer of crystalline powder spread on a BaF_2_ window. **Er**_**2**_ and **ErY@Y**_**2**_ samples were packed in the cryostat in an inert atmosphere and measured in this manner to avoid damage from contact with water and oxygen. A comparison of the IR spectra of **Er**_**2**_, **ErY@Y**_**2**_ and helicene-1,2-dione shows that two strong bands at 1682 and 1664 cm^–1^ disappear after the reaction of helicene-1,2-dione with Er­(BHT)_3_. These two bands are assigned to the C=O vibration of the ortho-quinone.^79^ The fingerprint region of the spectra is more difficult to interpret. After the reaction, strong bands appear at 1463, 1417, 1298, and 1274 cm^–1^. Strong bands at 1300–1200 cm^–1^ can be assigned to C–O vibrations of both helicene-1,2-diol and BHT ligands. For **Er**_**2**_ and **ErY@Y**_**2**_ there are also three strong bands in the region of 3000–2840 cm^–1^ which are absent for helicene-1,2-dione. These bands indicate C–H stretching vibrations of *tert*-butyl and methyl groups of the BHT ligand and C–H stretching vibrations in THF.

### Direct Current Magnetic Properties

The static magnetic behavior of the samples was investigated by direct current (DC) magnetic measurements using a SQUID magnetometer. The reported molar magnetic susceptibility and molar magnetization values are calculated per mole of Er^III^. The percentage of the erbium incorporated in the diamagnetically diluted sample **ErY@Y**_**2**_ was also estimated based on these data. The DC magnetic properties of **Er**_**2**_ and **ErY@Y**_**2**_ are presented in Figures 2 and 3 as field dependence of the molar magnetization and as temperature dependence of the product of molar magnetic susceptibility and temperature.

**Figure 2:**
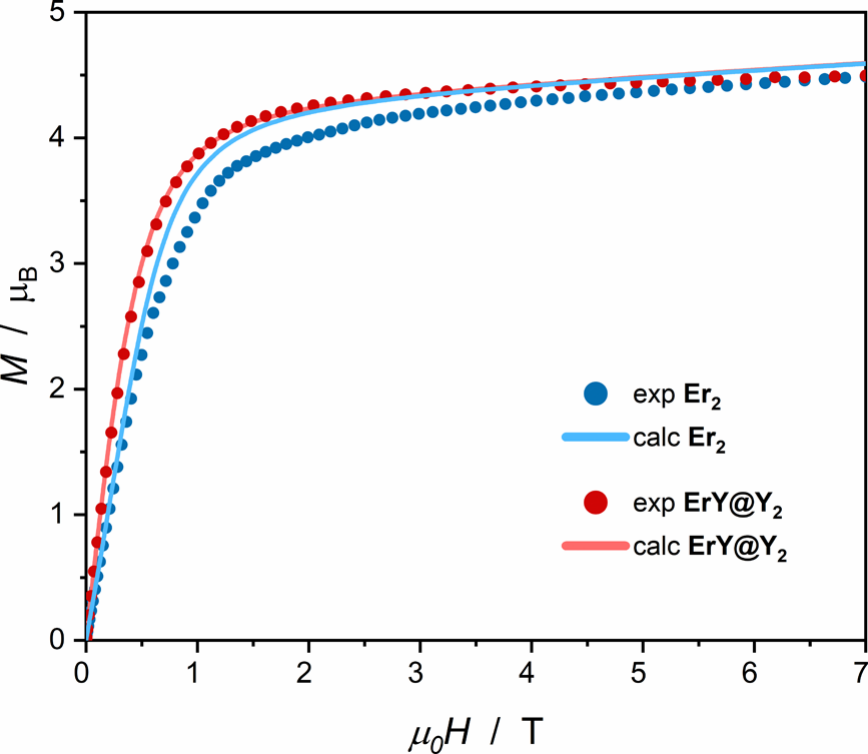
Comparison of the field dependence of the magnetization for **Er**_**2**_ (blue points; 1.8 K) and **ErY@Y**_**2**_ (red points; 2.0 K) calculated per mole of Er^III^. The CASSCF calculated curves for Er^III^ in mixed Er–Y dimers are simulated assuming no magnetic interactions (red line) while those for Er^III^ in the actual Er_2_ dimers are simulated with weak intramolecular antiferromagnetic interactions (*J* = −0.3 cm^–1^, *zJ* = −0.1 cm^–1^; blue line).

**Figure 3:**
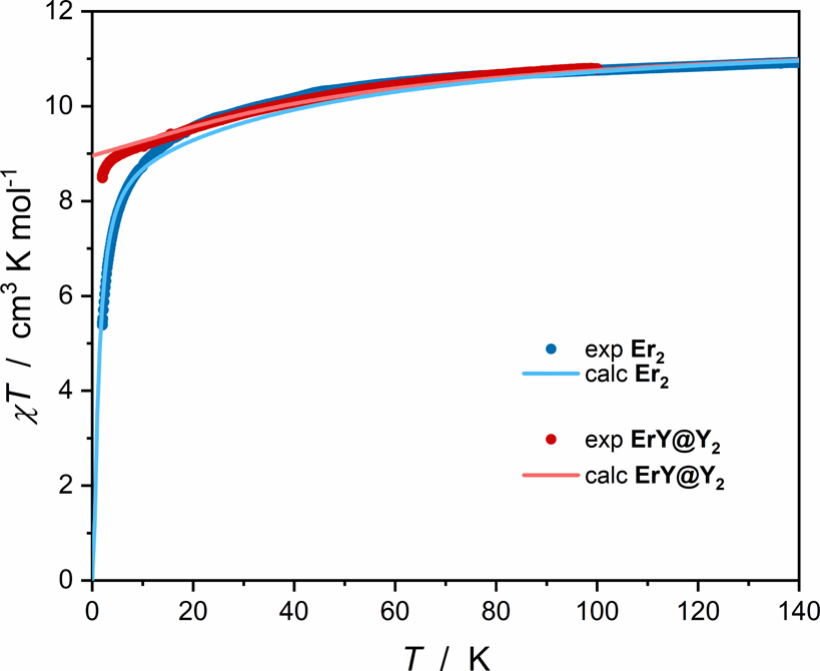
Temperature dependence of the magnetic susceptibility and temperature product for **Er**_**2**_ (blue circles) and **ErY@Y**_**2**_ (red circles) calculated per mole of Er^III^ in an applied magnetic field of 0.100 T in the range 2.0–100 K. Solid lines represent the CASSCF calculations simulated with weak intramolecular antiferromagnetic interactions within the dimer (*J* = −0.3 cm^–1^, *zJ* = −0.1 cm^–1^; blue line) or without any magnetic interactions (red line) for mixed-metal Er–Y solid state dilution.

The experimental value of the molar magnetization at 7 T and 2.0 K for **Er**_**2**_ is 4.49 μ_B_ per Er^III^ center (Figure 2), while the theoretical value for the free Er^III^ ion is 9.0 μ_B_.^80^ The reason that the experimental value does not reach saturation in the high magnetic field is the depopulation of the excited states due to *m*_J_ states splitting in the ligand field. The percentage of the Er^III^ in the **ErY@Y**_**2**_ diamagnetic dilution was determined to be 9.4% assuming that the saturation value of the magnetization at 7 T for **ErY@Y**_**2**_ should be the same as for the undiluted **Er**_**2**_ per Er^III^ center (4.49 μ_B_ at 7 T). This is in good agreement with the 10% used in the synthesis. Separate *M*(*H*) plots for **Er**_**2**_ and **ErY@Y**_**2**_ are presented in the SI (Figures S8 and S9).

The experimental *χT* values at 100 K of 10.76 cm^3^ K mol^–1^ for **Er**_**2**_ and 10.79 cm^3^ K mol^–1^ for **ErY@Y**_**2**_ (Figure 3), calculated per Er^III^ ion, are in good agreement with the free ion approximation of 11.48 cm^3^ K mol^–1^.^80^ Measurements up to room temperature were not possible due to the melting of the mother solution (142 K) in which the samples were immersed to prevent their structural damage. The clear decrease of the *χT* product on cooling is expected for lanthanide­(III)-based SMMs due to the thermal depopulation of the *m*_J_ states. However, below 8 K the decrease of the *χT* product is steeper for **Er**_**2**_ than for **ErY@Y**_**2**_. This can be explained by intramolecular antiferromagnetic interactions, which are present in the case of **Er**_**2**_ and almost absent in the case of **ErY@Y**_**2**_, due to the diamagnetic dilution - the statistical probability of finding an Er_2_ dimer in **ErY@Y**_**2**_ is only 1%.

### Alternating Current Magnetic Properties

Alternating current (AC) magnetic susceptibility measurements were performed for both samples, **Er**_**2**_ and **ErY@Y**_**2**_, to compare the magnetic dynamic behavior with and without Er–Er exchange interactions within the dimer. The frequency dependence of the AC magnetic susceptibility of **Er**_**2**_ at 1.8 K shows quite fast relaxation of the magnetization in the absence of the external magnetic field with the maximum of χ″ slightly above 1000 Hz (Figure S10, Table S4). After applying a small external DC field, the relaxation slows down with the longest relaxation times τ at the magnetic field within 600–800 Oe range. With the stronger DC field, the relaxation becomes faster again, which is common for field-induced SMMs, and the maximum of χ″ also becomes broader, as the second relaxation process starts to play a significant role. The sum of two modified Debye functions had to be used to fit the data over the whole range of magnetic fields, despite the fact that the **Er**_**2**_ molecule contains two structurally identical metal centers.^81^ A rather abnormal behavior is observed around the *H*_DC_ of 1800–3400 Oe, with the minimum in τ­(*H*) at about 2200 Oe and the second maximum at about 4000 Oe (dark blue points in Figure 4). This type of behavior is not often reported for field-induced SMMs.^82–84^ The reason may be that the magnetic field dependence of the AC susceptibility is generally less often studied than the temperature dependence of τ or simply the SMM relaxes at zero *H*_DC_ magnetic field.^85,86^ A similar case, but with τ reaching a plateau instead of decreasing at some intermediate values of *H*_DC_, was presented for the Yb­(trensal) complex and was explained by the phonon-bottleneck effect.^87^ In that case, this effect disappeared at slightly higher temperature. Hence, we decided to check the field dependence of the AC magnetic susceptibility at higher temperature as well (Figures S12 and S14; Tables S5 and S6). The τ­(*H*) dependence remains qualitatively the same also at 3.0 and at 4.5 K, with the same minimum at 2200 Oe and an additional maximum around 4000 Oe (Figure 4). The only difference is that at 4.5 K the slower process disappears and only a single relaxation process is sufficient for fitting the magnetic susceptibility plots (single Debye process).

**Figure 4:**
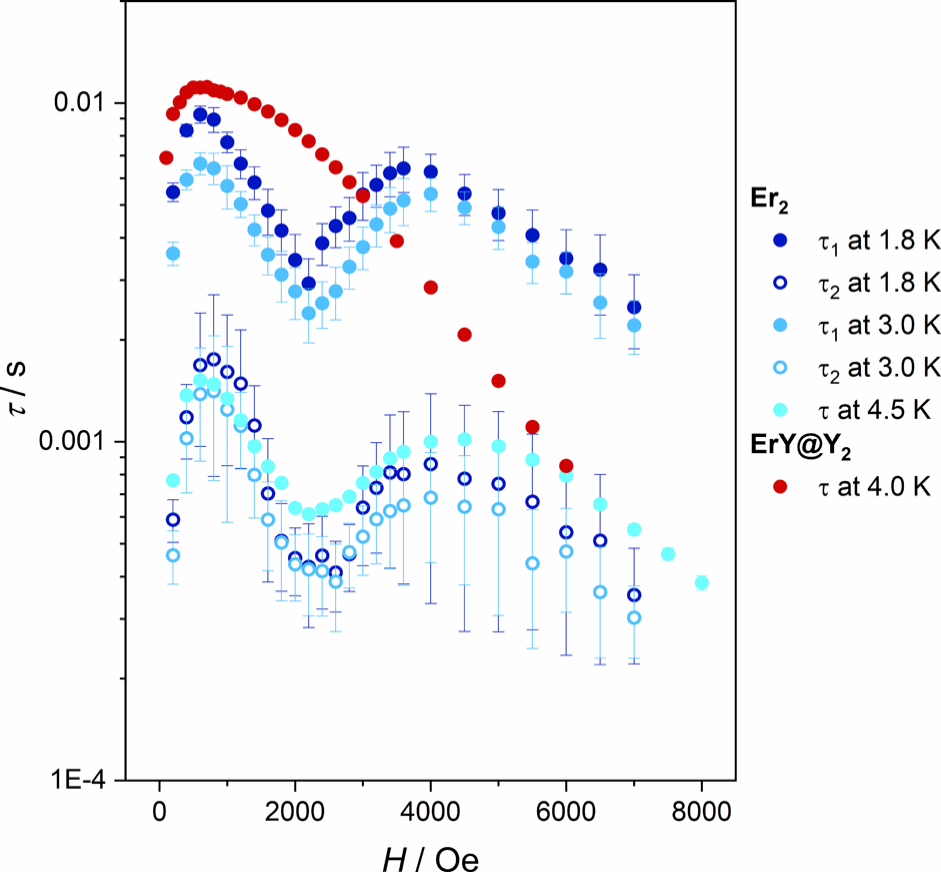
Field dependence of the relaxation time τ for **Er**_**2**_ at 1.8 K (dark blue points), 3.0 K (light blue points), and 4.5 K (cyan points) compared with field dependence of τ for **ErY@Y**_**2**_ at 4.0 K (red points). For **Er**_**2**_ data were described by two processes, longer τ_1_ (full circles) and shorter τ_2_ (open circles).

Equation 1 was used to fit the field dependence of the slow magnetic relaxation time τ extracted from the χ′,χ″(ν) dependencies:^88^

1 $$\tau \left(H\right)=1/\left(B_{1}/H^{2}+B_{2}H^{4}+B_{3}\right)$$

where the first part is a contribution of the quantum tunneling of magnetization (QTM), the second one stands for the direct relaxation process for Kramer’s ions and the constant *B*_3_ is related to the contribution of the field independent processes (Orbach and Raman).

The τ­(*H*) dependence extracted from the χ′,χ″(ν) data at each temperature (1.8 K, 3.0 K, 4.5 K) was fitted with eq 1 separately in the low magnetic field range (200–2200 Oe) and in the high magnetic field range (2200–7000 Oe or 2200–8000 Oe). These fits and parameters are presented in Figures S11, S13, S15 and Table S8 in the Supporting Information.

Diamagnetic dilution simplifies and slightly slows down the slow magnetic relaxation of the Er^III^ centers (Figure 4). The frequency dependence of the AC magnetic susceptibility (χ′,χ″(ν)) of **ErY@Y**_**2**_ has been measured at 4 K in the range 1–1000 Hz. The dynamic magnetic behavior of **ErY@Y**_**2**_ in the absence of an external magnetic field is very similar to that of undiluted **Er**_**2**_ – both samples show quite fast relaxation with the maximum of χ″ above 1000 Hz. In the case of **ErY@Y**_**2**_, a low magnetic field is required to significantly slow down the magnetic relaxation. At 100 Oe the maximum of χ″ is already around 23 Hz (Figure S16, Table S7). For the external magnetic field in the range of 400–800 Oe an identical relaxation is observed with the maximum of χ″ at about 14 Hz. For stronger *H*_DC_ fields, the relaxation gradually speeds up, but the second relaxation mechanism does not appear, in contrast to the **Er**_**2**_ relaxation at high *H*_DC_ fields. The AC data for **ErY@Y**_**2**_ were fitted using a Debye model for a single relaxation process. The τ­(*H*) dependence extracted from the χ′,χ″(ν) data was described by eq 1. The fit and parameters are shown in Figure S17 and Table S8 in the Supporting Information.

The temperature dependence of the AC magnetic susceptibility was measured at a DC field of 700 Oe for **Er**_**2**_ (Figure S18 and Table S9) and for **ErY@Y**_**2**_ (Figure S19 and Table S10), as this field was determined to be optimal for both samples (from the field dependence of χ′,χ″(ν)). In the lower temperature range **ErY@Y**_**2**_ relaxes significantly slower than **Er**_**2**_ (Figure 5). Above 5 K the slow magnetic relaxation of both compounds becomes similar. The values of the relaxation time τ extracted from the temperature dependencies of χ′,χ″(ν) for **Er**_**2**_ and **ErY@Y**_**2**_ were fitted using eq 2:^89^

2 $$\ln\tau \left(T^{-1}\right)=\ln\left[{{(C_{0}+C}_{1}T+C_{2}T^{n})}^{-1}\right]$$

where the consecutive terms stand for contributions from QTM, direct process and Raman mechanism. QTM (quantum tunneling of magnetization) in an external magnetic field (*H*_DC_ = 700 Oe) is assumed to be blocked. The addition of the Orbach relaxation part does not allow to find rational parameters for this process and does not improve the fitting curves. Based on that the Orbach process was excluded from further consideration.

**Figure 5:**
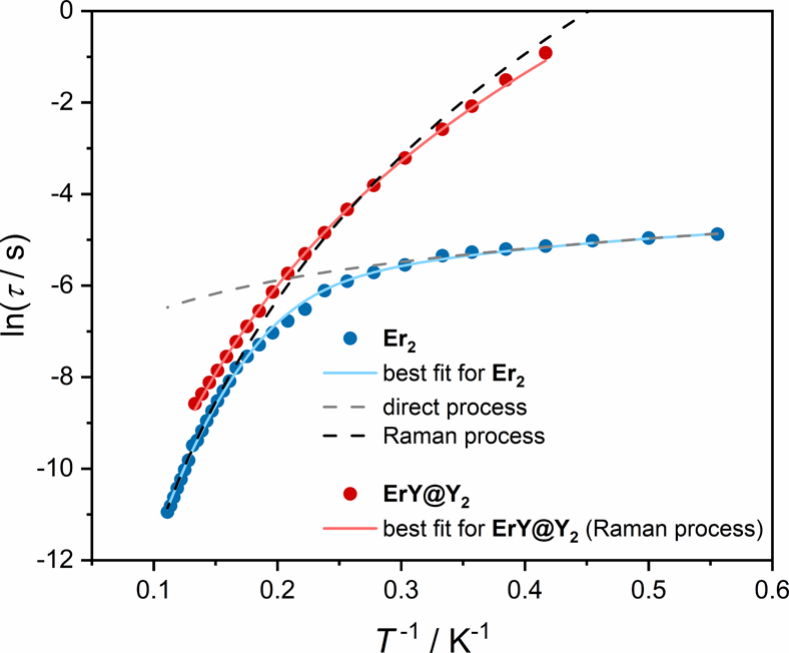
Comparison of temperature dependence of ln τ for **Er**_**2**_ and **ErY@Y**_**2**_ at 700 Oe.

The slow magnetic relaxation for **Er**_**2**_ in the low temperature range at 700 Oe is mainly driven by the direct process (*C*_1_ = 72(2) s^–1^ K^–1^). The Raman relaxation process starts to dominate above 5 K (*C*_2_ = 2.10(48) × 10^–3^ s^–1^ K^–1^, *n* = 7.75(12)). In the case of the diluted sample **ErY@Y**_**2**_, the data were fitted only with Raman process (*C*_2_ = 8.43(30) s^–1^ K^–1^, *n* = 6.69(2)) in the whole range of investigated temperatures (Figure 5, Table S11).

The slow magnetic relaxation in the absence of an external magnetic field was also analyzed for both samples. In both cases the maximum of χ″(ν) is visible between 1000 and 10000 Hz up to about 8.0 K (Figures S20 and S21; Tables S12 and S13). For **Er**_**2**_ the position of the maximum of χ″ does not change up to 5.0 K. For **ErY@Y**_**2**_ the position of the maximum is constant up to about 7.0 K. In both cases the intensity of the χ″ maximum decreases with increasing temperature, suggesting the QTM process as the main source of relaxation at zero magnetic field. The results of fitting the temperature dependence of lnτ confirm the influence of QTM and the direct process in both cases.

To summarize, the results of AC magnetic measurements show that diamagnetic dilution of the Er-based complex with yttrium ions efficiently eliminates Er–Er interactions in the dimers and leads to the formation of mixed ErY dimers in the Y_2_ matrix. Diamagnetic dilution eliminates the unusual τ­(H) dependence and slows down the magnetization reversal at lower temperatures (up to about 5 K; Figure 5). However, if QTM is not suppressed by the applied *H*_DC_ field, the undiluted **Er**_**2**_ sample relaxes slightly slower than **ErY@Y**_**2**_ in lower temperature regime (Figure 6; Table S14). Such a behavior might indicate that each second Er^III^ center in a the Er_2_ dimer is the source of a local magnetic field that efficiently suppresses the QTM in the undiluted **Er**_**2**_ compound – this effect is known as the exchange bias.^90,91^

**Figure 6:**
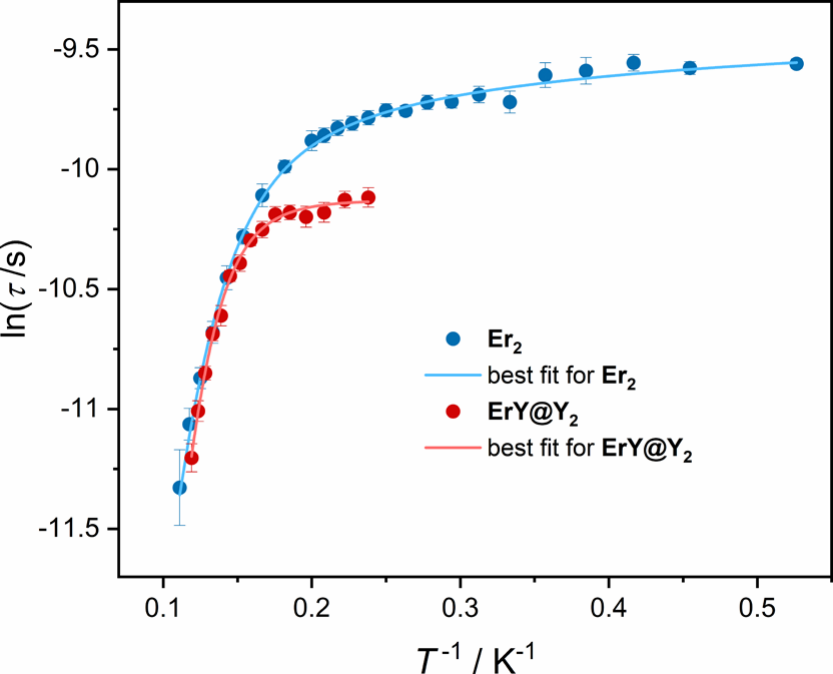
Comparison of temperature dependence of ln τ for **Er**_**2**_ and **ErY@Y**_**2**_ without applied *H*_DC_ field.

### Computational Analysis of the Influence of the Exchange Interaction on the Slow Relaxation of the Magnetization

The *ab initio* calculations for an isolated Er^III^ center in a **Er**_**2**_ dimer were performed using the crystal structure taken from the scXRD experiment without geometry optimization, assuming that the second center in the dimer is the diamagnetic Y^III^. The details can be found in the SI (Computational Details, Figure S22, Tables S15–S17). After obtaining the *ab initio* magnetic properties and orientation of the magnetization easy axis for a single Er^III^ ion in the molecule, POLY_ANISO module was employed to simulate the intramolecular magnetic interaction within the Er_2_ dimer. The simulations led to a clear conclusion that this interaction is in fact antiferromagnetic with *J*_Er–Er_ = −0.3 cm^–1^ (Lines model, see Figures 2 and 3 and details in the SI). This conclusion enables the understanding of the unusual τ­(*H*) dependence (Figure 7) extracted from the AC magnetic measurements. The simulated Zeeman splitting for the two lowest pseudodoublets of **Er**_**2**_ with Er^III^ centers coupled antiferromagnetically with *J*_Er–Er_ = −0.3 cm^–1^ (averaged over 17 directions over the hemisphere) shows the avoided level crossing exactly at *H*_DC_ = 2200 Oe (Figure 7), where the maximum in the relaxation rate is observed experimentally.

**Figure 7:**
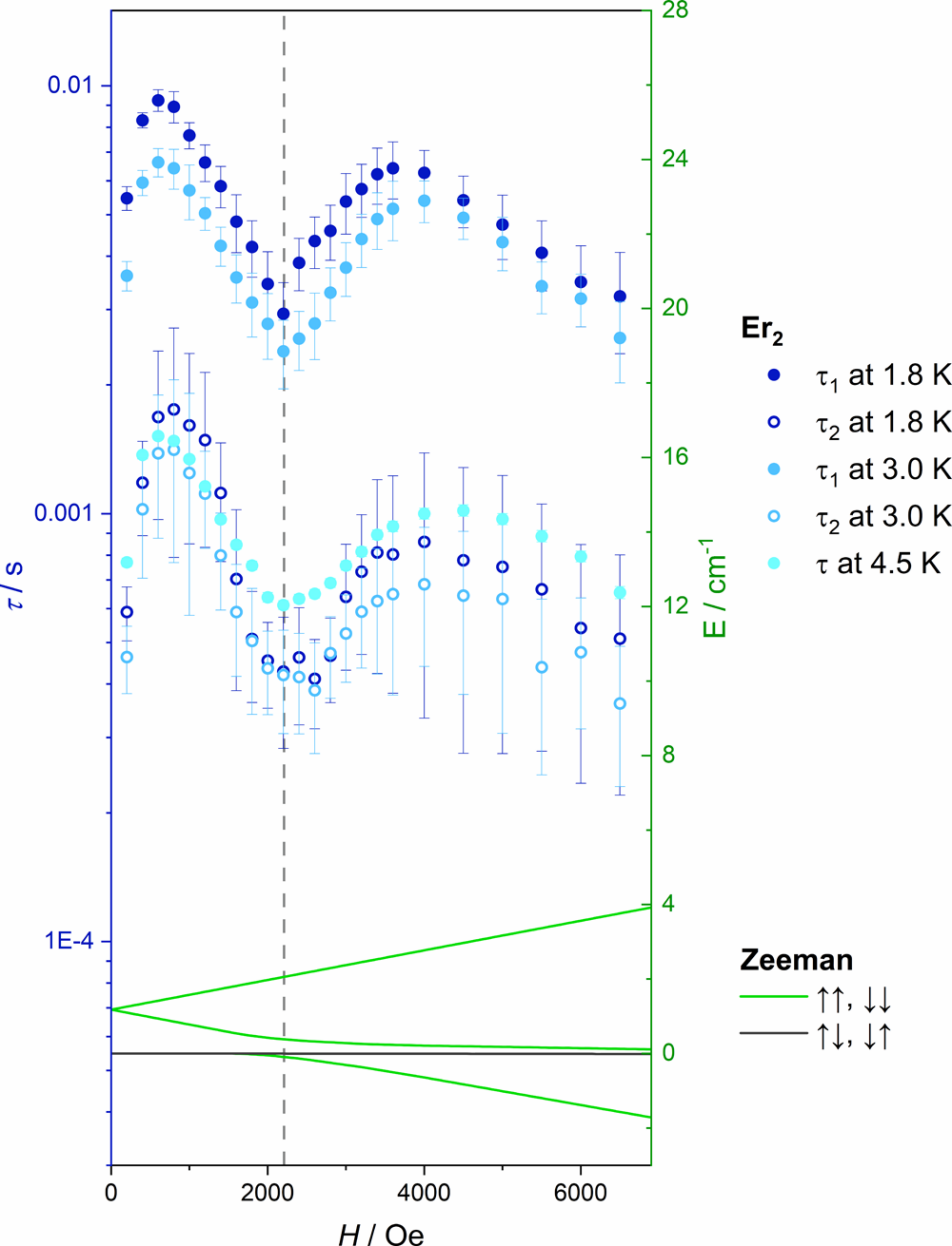
Comparison of the magnetic field dependence of the relaxation time τ for **Er**_**2**_ and the simulated Zeeman splitting for the two lowest pseudodoublets of **Er**_**2**_ explaining the maximum of the relaxation rate at 2200 Oe. The vertical gray dashed line indicates the magnetic field of avoided level crossing (*H*_cross_ = 2200 Oe) at which an efficient QTM process appears.

## Conclusions

A thorough magneto-structural characterization of the Er-based SMM (**Er**_**2**_) and its diamagnetic dilution with yttrium cations **ErY@Y**_**2**_ (where Er/Y 1:9) allowed the analysis of the erbium–erbium magnetic interactions in the dimer in the context of the slow magnetic relaxation of the whole molecule. It appears that the antiferromagnetic exchange coupling *J*_Er–Er_ in the **Er**_**2**_ dimer is the source of the peculiar relaxation time vs magnetic field dependence with the maximum of the relaxation rate occurring at the avoided level crossing (at *H*_cross_ = 2200 Oe; Figure 7) due to an efficient QTM process. The same feature is also visible in the DC magnetic measurements vs magnetic field in form of a butterfly-type magnetic hysteresis opening at the same *H*_cross_ magnetic field of 2200 Oe (Figure S8).

The next step of this work is to use pure helicene enantiomers to study the interplay of magnetism and chirality within a single molecule. Preparation of such magneto-chiral materials may allow the observation of second-order magneto-optical effects, like magneto-chiral dichroism or magnetic circularly polarized luminescence. Furthermore, it is noteworthy that even racemic forms of helicene-based molecules can exhibit intriguing properties. A recent study demonstrated that a purely organic molecule containing a helicene moiety exhibits notable spin-related properties associated with the photoinduced triplet state of the helicene core.^92^ Incorporating a helicene moiety as a bridge between two 4*f* metal ions may open new avenues for exploring the rich photophysics of helicenes.

## Experimental and Computational Methods

### Preparation of Compounds

Anhydrous chlorides of erbium(III) and yttrium­(III) (both 99.9% trace metal basis), and butylated hydroxytoluene (BHT; 98%) were purchased from commercial sources (Sigma-Aldrich or Alfa Aesar) and used as received. Sodium hydride was purchased from Sigma-Aldrich as a 60% dispersion in mineral oil and thoroughly washed with pentane before use. Er­(BHT)_3_ was synthesized according to the reported procedure^67^ and Y­(BHT)_3_ was synthesized analogously. [6]­Helicene-1,2-dione was synthesized according to the literature procedure.^68^ All operations were performed under an inert atmosphere (high purity Ar gas; O_2_ < 5 ppm; H_2_O < 0.1 ppm). The solvents used in the syntheses were dried using the Inert PureSolv EN7 solvent purification system and stored over 3 Å molecular sieves for at least 24 h before use to reduce the water content from around 20 ppm to a few ppm (4–6 ppm in the case of THF).^93^

### Synthesis of [Er­(hel)­(BHT)­(THF)_2_]_2_·2THF (Er_2_; H_2_hel = [6]­Helicene-1,2-diol)

[6]­Helicene-1,2-dione (0.083 mmol, 29.7 mg) solution in dry THF (3 mL) was added dropwise to Er­(BHT)_3_ (0.083 mmol, 68.3 mg) solution in dry THF (1 mL) with stirring. Yellow crystals were obtained by slow diffusion of dry pentane into the mother solution after 1 day. Slow diffusion of pentane is necessary to start crystallization, but it also results in crystallization of three different crystal phases of the complex (with different amounts THF and pentane molecules in the crystal structure). In order to obtain only one, pure crystal phase of this complex, the crystals were separated from the mother solution, crushed and mixed in a new portion of dry THF (about 5 mL) for 3 min and left for 3 days. After this time, only one crystal phase remained. Powder X-ray diffraction confirms the purity of the **Er**_**2**_ crystal phase after such procedure. Powder X-ray diffraction and magnetic measurements were performed for samples immersed in a small amount of the THF mother solution. After magnetic measurements, the sample was dried under active vacuum for 1 h to remove the crystallization solvent molecules (THF) from the structure and then weighed. The molar mass for the dried sample is 889.20 g/mol (calculated for the unit with one Er^III^ center, Er­(hel)­(BHT)­(THF)_2_).

### Synthesis of [Er_0.094_Y_0.906_(hel)­(BHT)­(THF)_2_]_2_·2THF (ErY@Y_2_; H_2_hel = [6]­Helicene-1,2-diol)

Solid state dilution of the erbium-based complex within the diamagnetic yttrium­(III) matrix was performed at the synthesis stage by mixing the appropriate amounts of Er­(BHT)_3_ (0.01 mmol, 8.3 mg) in dry THF (1 mL) and Y­(BHT)_3_ (0.09 mmol, 67.2 mg) in dry THF (1 mL). The solution of [6]­helicene-1,2-dione (0.1 mmol, 35.8 mg) in dry THF (3 mL) was added dropwise to this mixed solution of metal complexes while stirring. Crystallization and recrystallization were then carried out in a similar manner to **Er**_**2**_. The isostructural character of the obtained sample was confirmed by powder X-ray diffraction (Figure S6). The molar mass was calculated for the sample without any crystallization solvent molecules: 889.20 g/mol (for one Er^III^ center) and 810.85 g/mol (for one Y^III^ center). For mass determination, the sample was dried under active vacuum for 1 h. The percentage of Er^III^ in the **ErY@Y**_**2**_ sample was determined by matching the saturation magnetization value for **ErY@Y**_**2**_ (at 7 T) with the value measured for **Er**_**2**_ sample (Figure 2).

### Single Crystal X-ray Diffraction

Single crystal X-ray diffraction (scXRD) data were collected using a Bruker D8 Quest Eco Photon50 CMOS diffractometer equipped with a MoKα radiation source and graphite monochromator. Details of the measurements and refinements for **Er**_**2**_ (100 and 270 K) and **ErY@Y**_**2**_ (100 K) are given in Table S1. Unit cell refinement, data reduction, scaling and absorption corrections (multiscan) were performed using the SADABS and SAINT programs included in the Apex3 suite. Structures were determined by direct methods using Apex3 software (ShelXT) and refined anisotropically in OLEX2 (ShelXL) using weighted full-matrix least-squares on *F* ^2^.^94–96^ Hydrogen atoms were placed in calculated positions and refined by applying riding model. Crystal structures figures were generated using Mercury CSD 4.0.^97^

### Powder X-ray Diffraction

Powder X-ray diffraction (pXRD) measurements were performed at room temperature using a Bruker D8 Advance Eco diffractometer equipped with a CuKα radiation source and a graphite monochromator. Samples were finely ground under mother liquor and then loaded into 0.5 mm diameter glass capillaries with a small amount of solution to prevent desolvation.

### IR Spectra

IR spectra were recorded using a Nicolet iN10 MX FT-IR microscope in the transmission mode. A small amount of powdered sample was spread on the BaF_2_ pellet in a glovebox and placed in an airtight cryostat prior to measurement to prevent contact of the sample with air and moisture.

### Magnetic Measurements

Direct current (DC) measurements for both samples were performed using a Quantum Design MPMS-3 Evercool magnetometer under magnetic fields up to 7 T. Alternating current (AC) magnetic measurements were performed using a Quantum Design MPMS-3 Evercool magnetometer (in the frequency range 1–1000 Hz) and also using a Physical Property Measurement System PPMS (in the frequency range 10–10000 Hz). Each sample was ground to powder under mother solution, loaded into the glass tube (5 mm in diameter) with a minimal amount of mother solution in glovebox, cooled in liquid nitrogen at the Schlenk line and the tube was closed by melting the glass while applying vacuum. In this way, the samples were protected from water and oxygen at all times. The glass tube was then placed in a long plastic straw with an additional empty glass tube below the tube containing the sample. The samples were cooled to 140 K (below the freezing point of the THF) in the absence of the magnetic field to avoid the orientation of the crystallites. The experimental results were corrected for the diamagnetism of the sample itself and the sample holder. The mass of each sample was determined after magnetic measurements after drying the sample for 1 h under active vacuum. The dried sample was weighed in a glovebox to avoid contact with air. All magnetic data were calculated per one Er^III^ center using the molar mass of half of the **Er_2_** molecule: Er­(hel)­(BHT)­(THF)_2_.

### Computational Methods

*Ab initio* calculations of the magnetic properties of a single Er^III^ ion in the **Er**_**2**_ dimer were performed using the crystal structure model from the scXRD experiment without geometry optimization, assuming that the second center in the dimer is the diamagnetic Y^III^ ion. Subsequently, the POLY_ANISO module was used to simulate the intramolecular magnetic interaction within the **Er**_**2**_ dimer. Complete computational details can be found in the Supporting Information.

## Supplementary Material



## Notes

Synthetic details, experimental methods, single crystal X-ray diffraction, powder X-ray diffraction, infrared spectroscopy, magnetic measurements, computational details, references (PDF)
