# Supplementary material for: Exchange Bias in a Dinuclear Erbium Single-Molecule Magnet Bridged by a Helicene Ligand
Source: Inorg Chem. 2025 Jul 17;64(29):15088–97. doi: 10.1021/acs.inorgchem.5c01992 (PMC12308793; doi:10.1021/acs.inorgchem.5c01992)
Supplement: Supplementary file 1 [file ic5c01992_si_001.pdf]

SUPPORTING INFORMATION: Exchange Bias in a Dinuclear Erbium Single-Molecule Magnet Bridged by a Helicene Ligand

Gabriela Handzlik [a], Mikołaj Żychowicz [a], Katarzyna Rzepka [a], Dawid Pinkowicz [a]**

[a] Faculty of Chemistry, Jagiellonian University, Gronostajowa 2, 30-387 Kraków, Poland

*Email: gabriela.handzlik@uj.edu.pl

*Email: dawid.pinkowicz@uj.edu.pl

Table of contents

SINGLE CRYSTAL X-RAY DIFFRACTION 3

POWDER X-RAY DIFFRACTION 9

INFRARED SPECTOSCOPY 10

MAGNETIC MEASUREMENTS 11

Static magnetic properties 11

Dynamic magnetic properties 13

COMPUTATIONAL DETAILS 35

REFERENCES 41

SINGLE CRYSTAL X-RAY DIFFRACTION

Table S1. Crystal structure solution and refinement parameters for **Er_2** (at 100 K and at 270 K) and **ErY@Y_2** (at 100 K).

Formula	$C_{106}H_{122}Er_2O_{12}$		$C_{106}H_{122}Er_{0.188}O_{12}Y_{1.812}$
M_r/g mol^{-1}	1922.55		1780.74
T/K	100	270	100
CCDC	2331535	2331537	2331536
Crystal system	Monoclinic		
Space group	$P2_1/c$		
a/Å	13.4620(8)	13.6312(9)	13.4541(15)
b/Å	15.0525(8)	15.3221(11)	15.0598(13)
c/Å	22.3890(12)	22.4671(12)	22.3719(18)
β/°	107.300(3)	107.285(4)	107.335(4)
V/Å^3	4331.6(4)	4480.5(5)	4327.0(7)
Z	2	2	2
ρ_{calc}/g cm^{-3}	1.474	1.425	1.367
μ/mm^{-1}	1.99	1.92	1.46
F(000)	1980	1980	1875
Crystal size/mm^3	0.4 x 0.05 x 0.04		0.38 x 0.07 x 0.06
Radiation	Mo Kα (λ = 0.71073 Å)		
2θ range/°	2.7-26.8	2.8-27.1	2.5-26.0
Reflections collected	57913	8452	49554
Independent reflections	9194	6001	8506
R_{int}	0.067	0.037	0.063
restrains/parameters	115/534	75/531	75/537
$R[F_o>2\sigma(F_o)]$	0.069	0.054	0.070
$wR(F^2)$	0.153	0.118	0.186
GOF on F^2	1.152	1.034	1.031
$\Delta\rho_{max}$, $\Delta\rho_{min}$ /e Å^{-3}	2.51, -2.08	1.12, -0.61	1.78, -1.29
Completeness/%	99.9	64.0	99.8

The increase of the temperature from 100 K to 270 K changes the volume of the **Er_2** unit cell by +3.4% (Table S1). The unit cell of **ErY@Y_2** at 100 K is almost identical to that of **Er_2** at the same temperature. The low completeness of the data for the structure measured at 270 K is due to the need for a short measurement - the crystal easily loses THF crystallization molecules at this relatively high temperature. Even in the crystal structure measured at 100 K, the crystallization THF molecule is slightly disordered and has been refined with enlarged ellipsoids of carbon and oxygen atoms. Therefore, it was modeled by implementing the THF molecule from the Fragment Database. Due to the easy desolvation of the crystals, powder X-ray diffraction and magnetic measurements were performed under a small amount of mother solution. One of the coordination THF molecules also has some freedom of movement. It was modeled as disordered between two positions. The occupancies of these two positions were left free during the refinement. They are close to 0.5 but differ slightly in three crystal structures: 0.491 and 0.509 for **Er_2** at 100 K, 0.448 and 0.552 for **Er_2** at 270 K and 0.497 and 0.503 for **ErY@Y_2** at 100 K. The occupancy of the metal center position in **ErY@Y_2** was fixed according to the results of magnetic measurements as 9.4% Er and 90.6% Y, which is consistent with the amounts of metal precursors used in the synthesis (10% of Er(BHT)$_3$ and 90% of Y(BHT)$_3$).

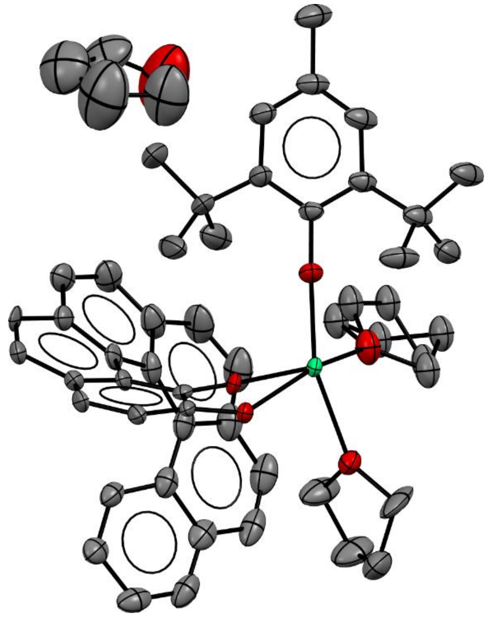

Figure S1. The asymmetric unit of the **Er_2** dimer (at 100 K). Ellipsoids are shown at the 50% probability level. Green – erbium, gray – carbon, red – oxygen; hydrogen atoms are omitted for clarity.

Table S2. Analysis of the coordination geometry of Er1 with Shape v2.1 (continuous shape measure analysis,[1] ideal geometry would give a value equal to 0).

Coordination geometry	Value from Shape v2.1
Octahedron, Oh	5.393
Trigonal prism, D3h	9.175
Pentagonal pyramid, C5v	18.302
Johnson pentagonal pyramid J2, C5v	22.719
Hexagon, D6h	32.499

Table S3. Analysis of Er-O bond lengths in $\mathbf{Er_2}$ at 100 K. Bonds are ordered from the shortest to the longest.

Bond	Bond length / Å
Er1-O3 (oxygen atom from BHT)	2.112(4)
Er1-O2 (non-bridging oxygen atom from helicene)	2.176(5)
Er1-O1 (O1 from other asym. unit)	2.273(5)
Er1-O1 (O1 from the same asym. unit)	2.283(5)
Er1-O5 (oxygen atom from disordered THF)	2.364(8)
Er1-O4 (oxygen atom from non-disordered THF)	2.375(4)

Hydrogen interactions

The crystal structure of the dimer is supported by an array of weak hydrogen bonds. The oxygen atom (O6) of crystallization THF molecule is 2.841 Å away from the nearest hydrogen atom (H3 in the helicene ligand), which can be considered a very weak hydrogen bond with the C3-H3-O6 angle of 137.9° and the C3-O6 distance of 3.60 Å (Figure S2).[2]

Another weak interactions can be found between the hydrogen atoms of the THF coordination molecule and the centroids of the aromatic rings of the helicene of the neighboring dimer. The distance between H43A and the centroid of the fifth ring (C13 C14 C15 C16 C21 C22) is 2.793 Å and the distance between H44B and the centroid of the sixth ring (C16 C17 C18 C19 C20 C21) is 2.921 Å (Figure S3). The distances between the carbon atoms of this THF molecule and the centroids of the aromatic rings are 3.718 Å and 3.769 Å, respectively. The C-H-(centroid) angles for these interactions are equal to 155.65° and 144.24°, respectively.

The second coordinated THF molecule in the asymmetric unit (the disordered one) is oriented towards the BHT ligand of the neighboring dimer. Among the hydrogen atoms of the disordered THF coordination molecule, H48A is the closest to the centroid of the BHT aromatic ring (2.690 Å) (Figure S4). The corresponding C-H-(centroid) distance is equal to 3.474 Å and the C-H-(centroid) angle is 136.38°. The second position of this disordered THF molecule results in an even weaker interaction with the BHT ring.

The *tert*-butyl group of the BHT ligand is also stabilized by weak hydrogen interactions. In the same asymmetric unit, H36A is 2.537 Å away from the centroid of the third aromatic ring (C7 C8 C9 C10 C23 C24) of the helicene ligand and H35A is 2.873 Å away from the centroid of the second aromatic ring (C4 C5 C6 C7 C24 C25) of the helicene ligand (Figure S5). The C-H-(centroid) distances for these interactions are equal to 3.463 Å and 3.748 Å, respectively and the angles C-H-(centroid) are equal to 157.41° and 149.21°, respectively.

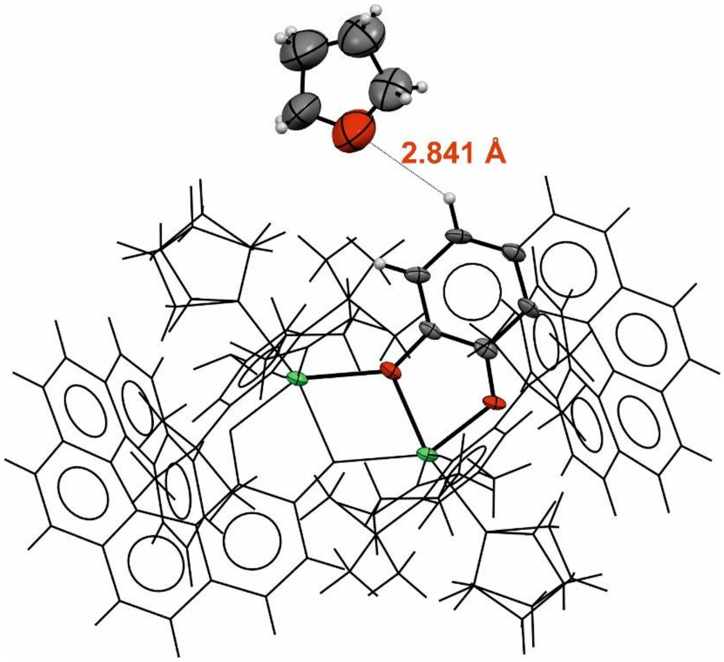

Figure S2. Weak hydrogen bond between crystallization THF molecule and the neighboring dimer.

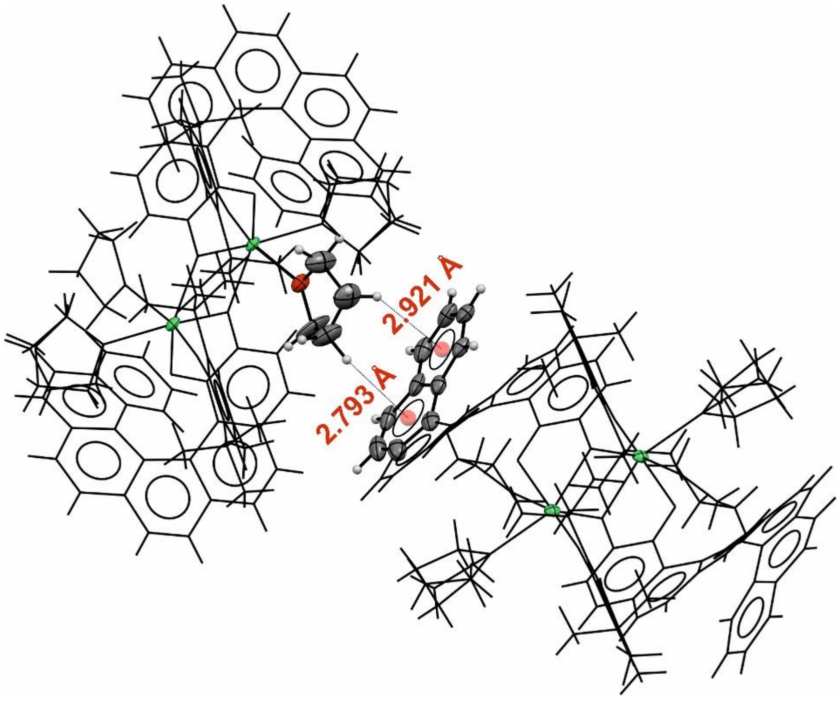

Figure S3. Weak hydrogen bond between *tert*-butyl group of BHT ligand and centroids of helicene aromatic rings from the neighboring dimer molecule.

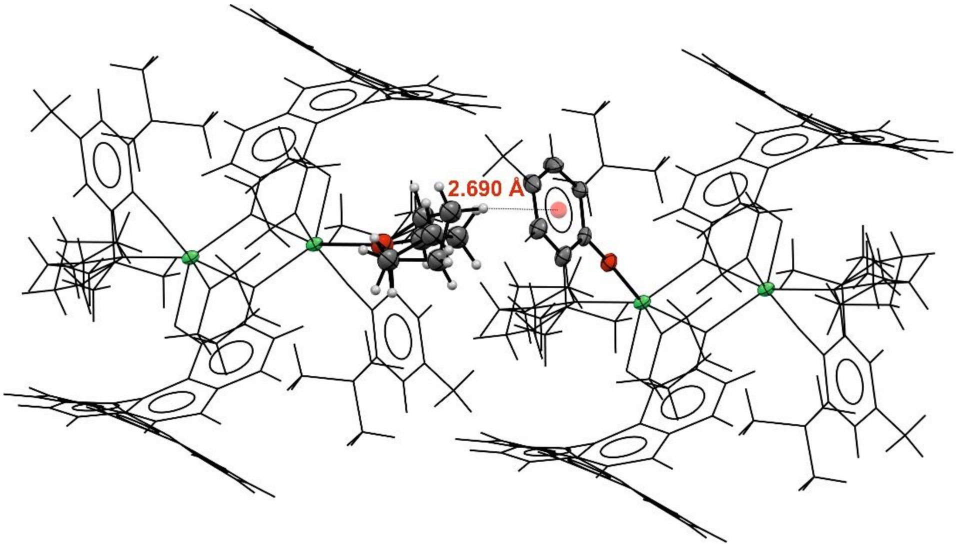

Figure S4. Weak hydrogen bond between *tert*-butyl group of BHT ligand and centroids of aromatic rings.

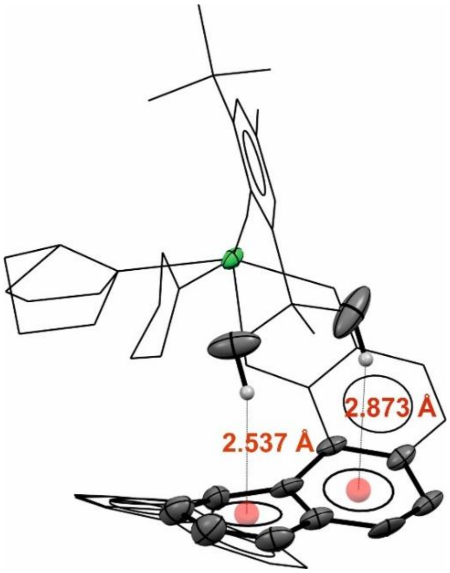

Figure S5. Weak hydrogen bond between *tert*-butyl group of BHT ligand and centroids of aromatic rings in the asymmetric unit.

POWDER X-RAY DIFFRACTION

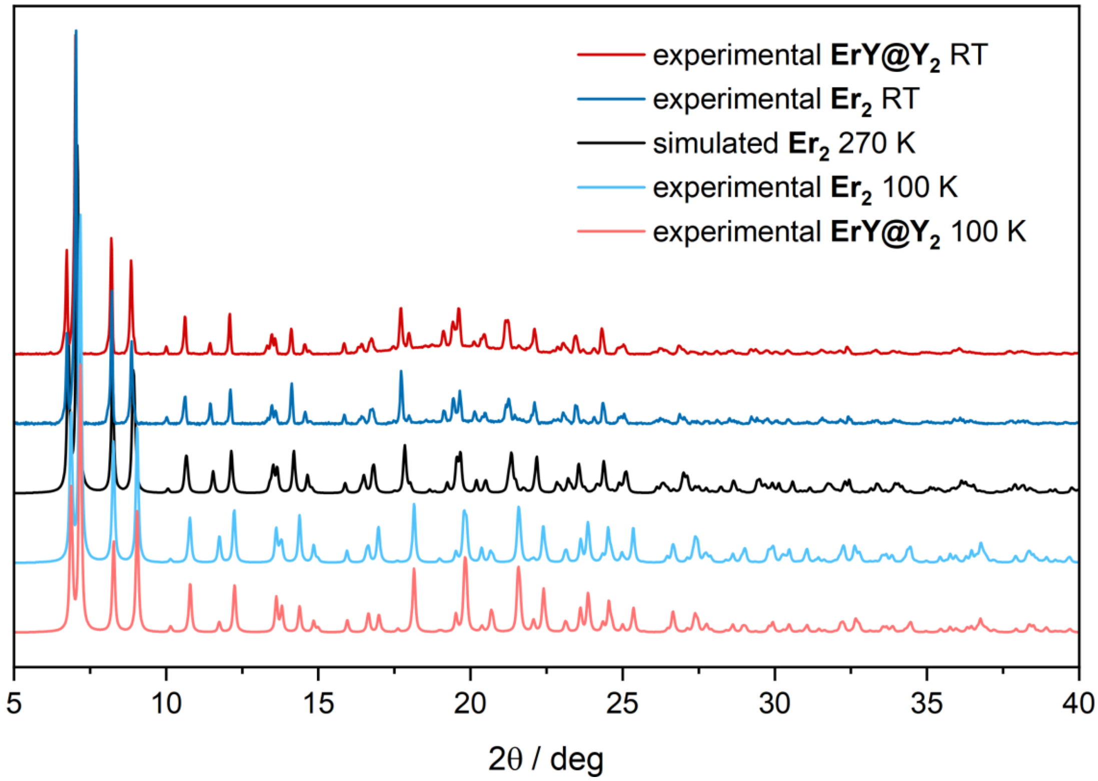

Figure S6. Comparison of the pXRD patterns simulated from the scXRD measurements with experimental pXRD patterns for $\mathbf{Er_2}$ and $\mathbf{ErY@Y_2}$ at room temperature.

INFRARED SPECTOSCOPY

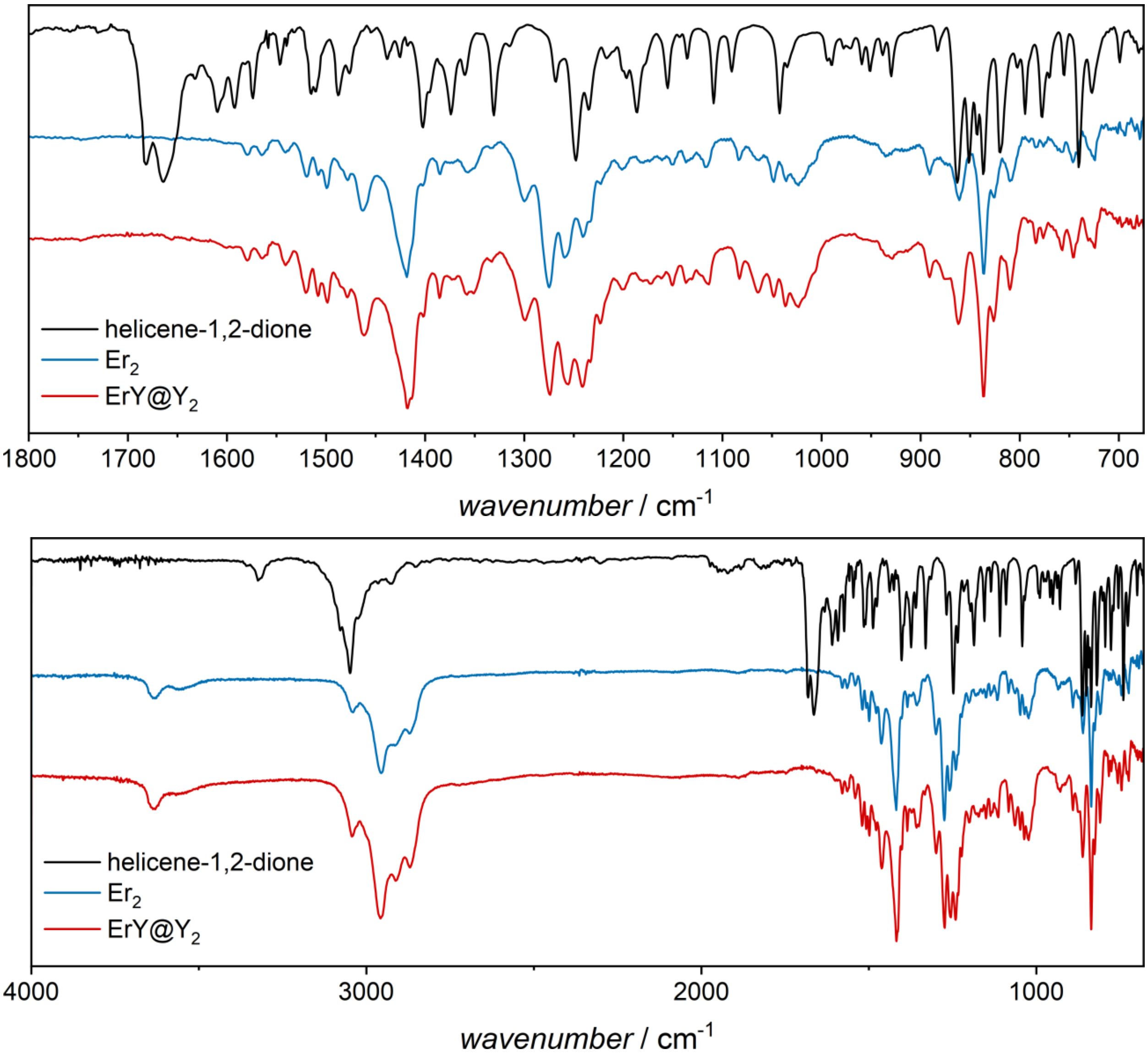

Figure S7. Comparison of the IR spectra of **Er_2** (blue line) and **$ErY@Y_2$** (red line) complexes and helicene-1,2-dione (black line).

MAGNETIC MEASUREMENTS

Static magnetic properties

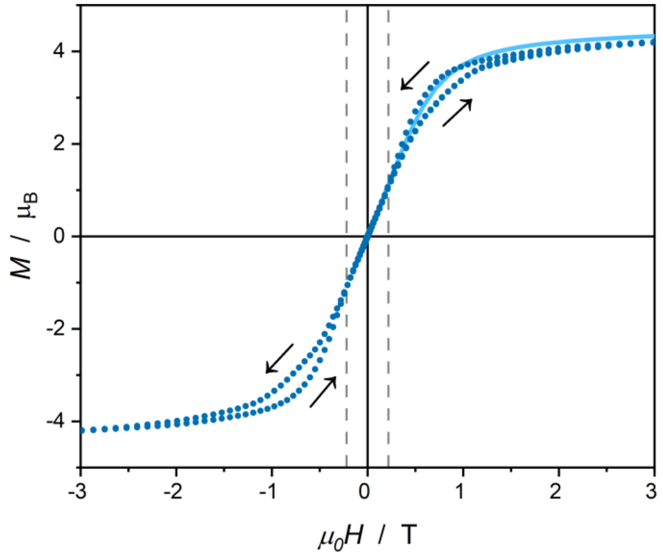

Figure S8. $M(H)$ dependence for **Er2** with butterfly-type magnetic hysteresis (blue points; T = 1.8 K; the change of the magnetic field direction is indicated with arrows). Hysteresis opens at the H_{cross} = 2200 Oe (gray dash lines) at which spins in the dimer change their relative orientation. Calculated curve was simulated with weak antiferromagnetic interactions between metal centers in the dimer (light blue line; J = –0.3 cm^{-1}, zJ = –0.1 cm^{-1}). The reported molar magnetization values are calculated per mole of Er^{III}.

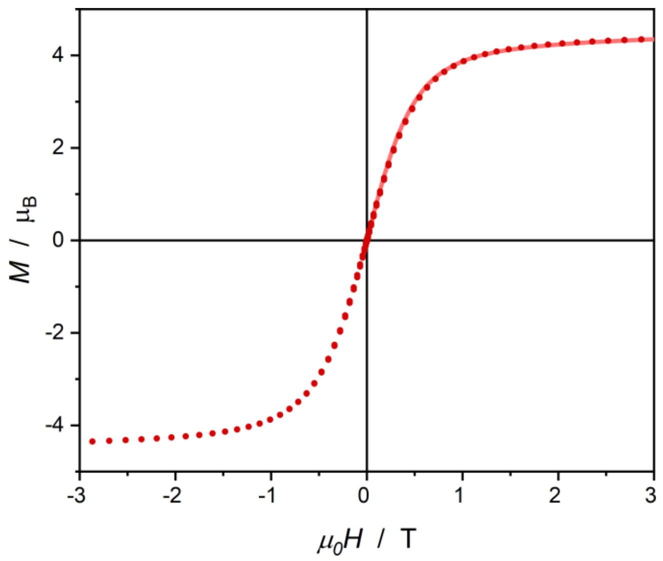

Figure S9. Magnetic field dependence of the magnetization for **ErY@Y$_2$** (red points; T = 2.0 K) and calculated curve simulated without metal-metal interactions (light red line). The reported molar magnetization values are calculated per mole of Er^{III}.

Dynamic magnetic properties

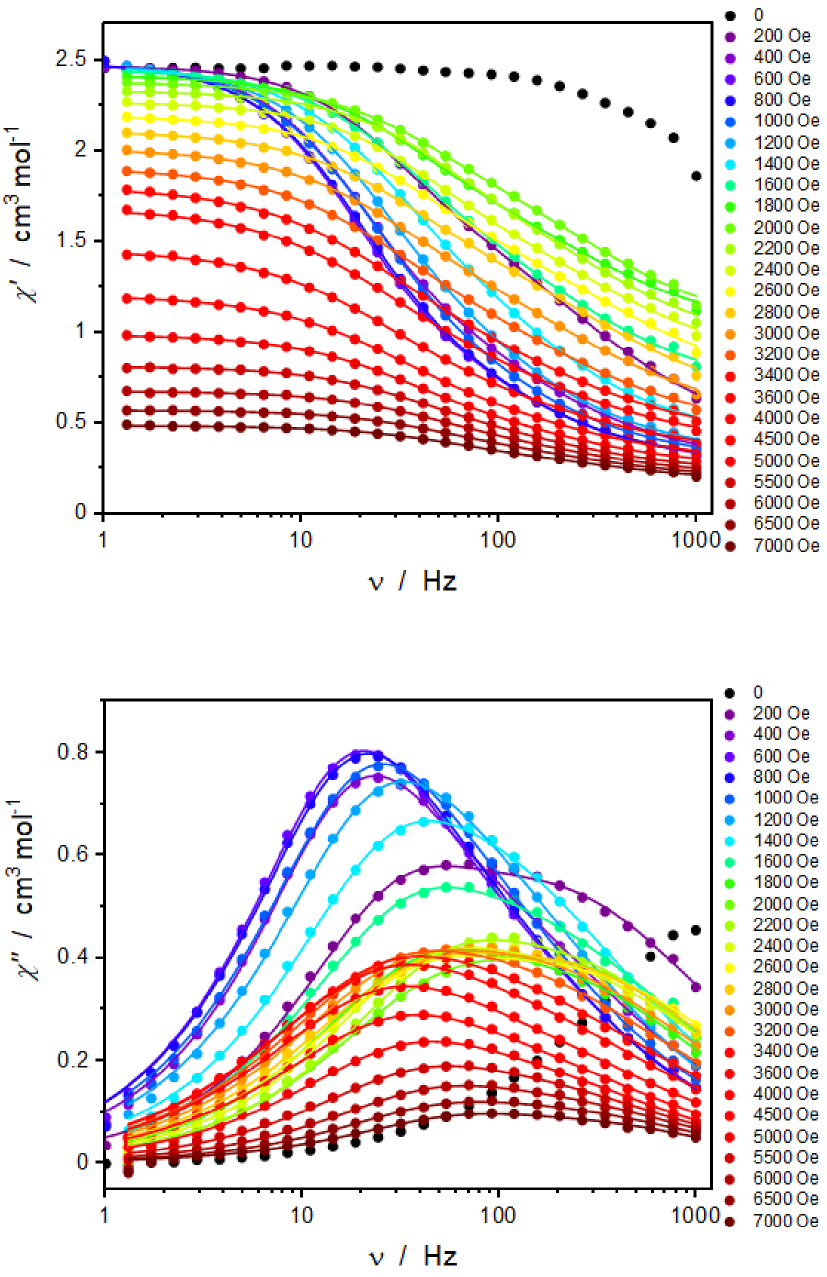

Figure S10. In-phase (χ') and out-of-phase (χ'') AC susceptibilities for **Er2** at 1.8 K measured in various fields H_{DC} (0-7000 Oe). Values of α and τ parameters are presented in Table S4.

Table S4. Values of α and τ from fitting two modified Debye functions to τ(ν) dependencies for **Er_2** at 1.8 K.

H **/ Oe**	**α_1**	**τ_1 / s**	**α_2**	**τ_2 / s**
200	0.06(3)	0.0055(3)	0.27(4)	0.00059(9)
400	0.06(2)	0.0083(3)	0.28(4)	0.0012(3)
600	0.07(3)	0.0093(5)	0.27(5)	0.0017(7)
800	0.08(4)	0.0089(8)	0.26(6)	0.0018(10)
1000	0.09(3)	0.0077(5)	0.28(4)	0.0016(8)
1200	0.10(4)	0.0066(6)	0.26(4)	0.0015(7)
1400	0.09(4)	0.0058(6)	0.25(4)	0.0011(3)
1600	0.11(6)	0.0048(7)	0.28(8)	0.0007(3)
1800	0.13(4)	0.0042(6)	0.21(9)	0.0005(1)
2000	0.14(4)	0.0034(6)	0.18(8)	0.0005(1)
2200	0.12(4)	0.0029(5)	0.19(11)	0.0004(1)
2400	0.11(5)	0.0039(5)	0.24(9)	0.0005(1)
2600	0.14(4)	0.0043(6)	0.26(8)	0.0004(1)
2800	0.16(4)	0.0046(7)	0.25(7)	0.0005(1)
3000	0.15(5)	0.0054(9)	0.28(7)	0.0006(2)
3200	0.17(4)	0.0057(8)	0.29(6)	0.0007(3)
3400	0.18(4)	0.0062(9)	0.31(8)	0.0008(4)
3600	0.18(4)	0.0064(10)	0.29(9)	0.0008(4)
4000	0.17(4)	0.0063(8)	0.31(8)	0.0009(5)
4500	0.17(4)	0.0054(7)	0.28(11)	0.0008(5)
5000	0.14(5)	0.0047(8)	0.24(12)	0.0008(5)
5500	0.12(5)	0.0041(8)	0.23(12)	0.0007(4)
6000	0.12(5)	0.0035(7)	0.20(15)	0.0005(3)
6500	0.11(7)	0.0032(9)	0.19(16)	0.0005(3)
7000	0.14(5)	0.0025(6)	0.08(21)	0.0004(1)

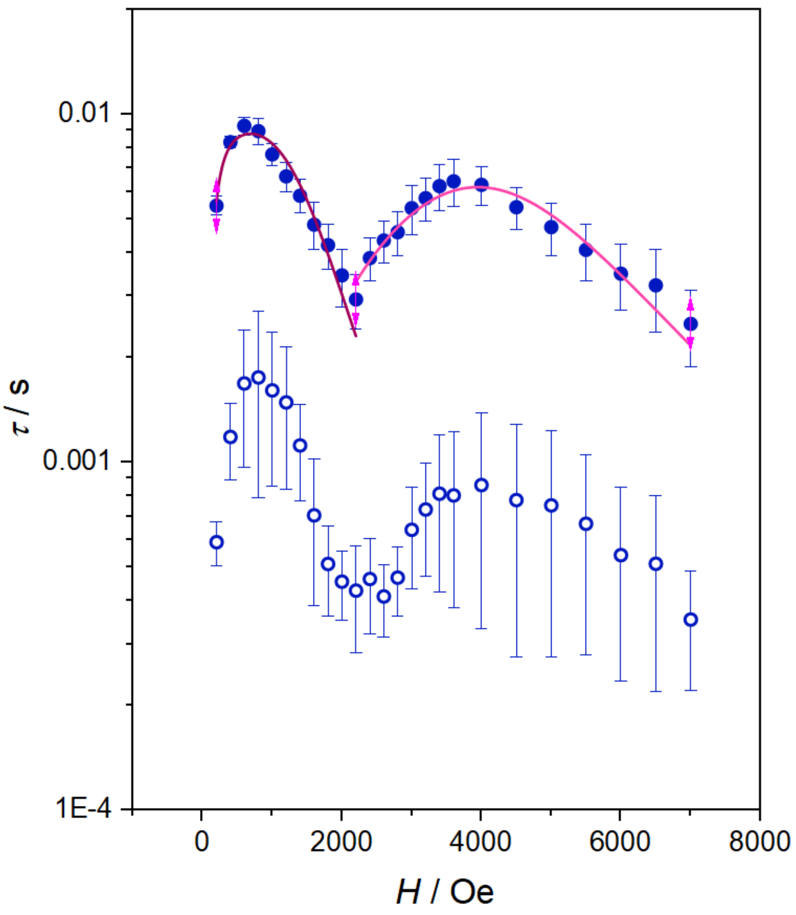

Figure S11. Magnetic field (H_{DC}) dependence of τ for **Er2** at 1.8 K described by two processes – longer τ_1 (full circles) and shorter τ_2 (open circles). Longer τ_1 was fitted with Equation 1 (main text) in two separate ranges. Details about the fits are collected in Table S8.

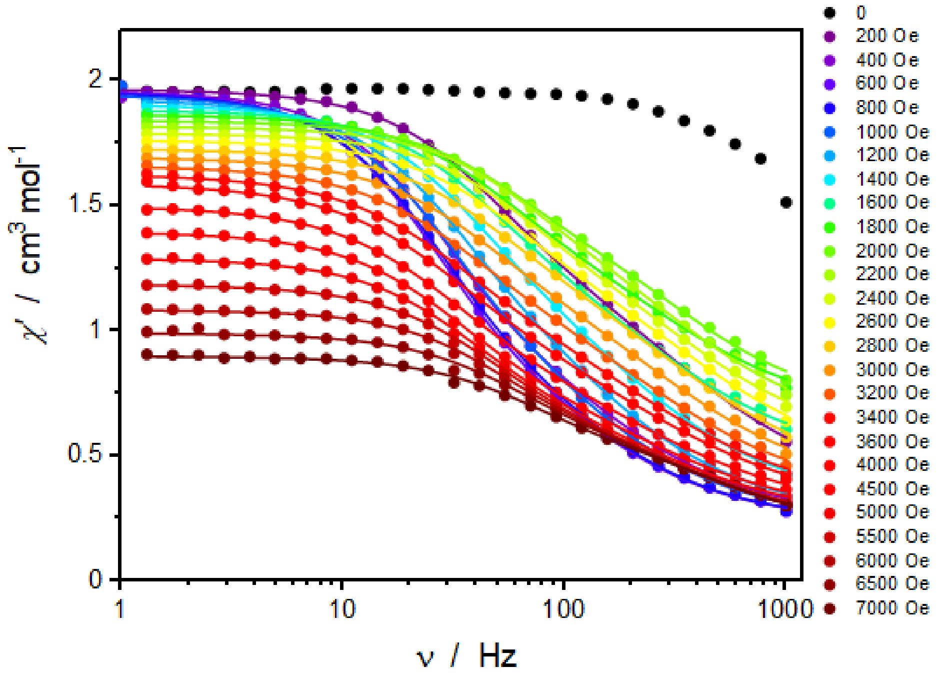

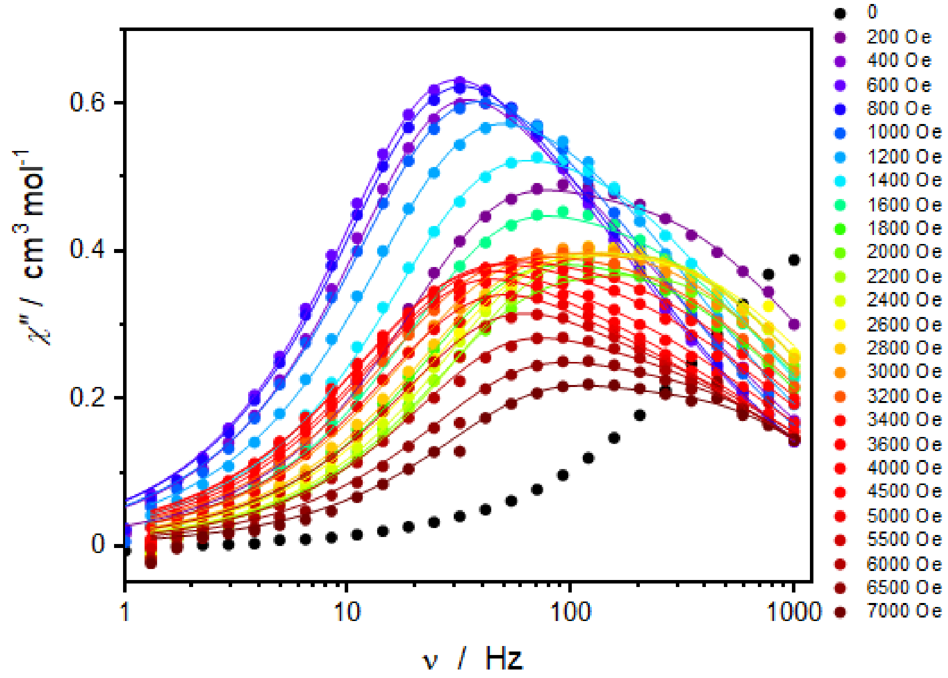

Figure S12. In-phase (χ') and out-of-phase (χ'') AC susceptibilities for **Er_2** at 3.0 K measured in various fields H_{DC} (0-7000 Oe). Values of α and τ parameters are presented in Table S5.

Table S5. Values of α and τ from fitting two modified Debye functions to τ(ν) dependencies for $\mathbf{Er_2}$ at 3.0 K.

H **/ Oe**	α_1	τ_1 / s	α_2	τ_2 / s
200	0.05(4)	0.0036(3)	0.24(5)	0.00046(8)
400	0.03(3)	0.0059(4)	0.23(5)	0.0010(3)
600	0.04(4)	0.0066(5)	0.22(5)	0.0014(5)
800	0.05(5)	0.0064(7)	0.22(6)	0.0014(6)
1000	0.06(6)	0.0057(8)	0.22(7)	0.0012(7)
1200	0.05(4)	0.0050(4)	0.23(3)	0.0011(3)
1400	0.05(5)	0.0042(4)	0.22(4)	0.0008(2)
1600	0.05(5)	0.0036(5)	0.21(7)	0.0006(2)
1800	0.06(6)	0.0031(5)	0.19(9)	0.0005(2)
2000	0.06(5)	0.0028(5)	0.15(8)	0.0004(1)
2200	0.05(6)	0.0024(4)	0.16(8)	0.0004(1)
2400	0.05(6)	0.0026(4)	0.18(8)	0.0004(1)
2600	0.08(6)	0.0028(5)	0.20(10)	0.0004(1)
2800	0.07(5)	0.0033(5)	0.20(6)	0.0005(1)
3000	0.08(5)	0.0038(5)	0.21(6)	0.0005(1)
3200	0.09(5)	0.0044(6)	0.23(7)	0.0006(2)
3400	0.11(5)	0.0049(7)	0.25(8)	0.0006(2)
3600	0.11(6)	0.0052(8)	0.27(9)	0.0006(3)
4000	0.09(5)	0.0054(6)	0.27(7)	0.0007(2)
4500	0.08(5)	0.0049(5)	0.26(9)	0.0006(3)
5000	0.07(6)	0.0043(6)	0.24(11)	0.0006(3)
5500	0.08(5)	0.0034(5)	0.21(14)	0.0004(2)
6000	0.05(5)	0.0032(4)	0.18(11)	0.0005(2)
6500	0.08(6)	0.0026(6)	0.13(17)	0.0004(1)
7000	0.08(4)	0.0022(4)	0.10(15)	0.00030(7)

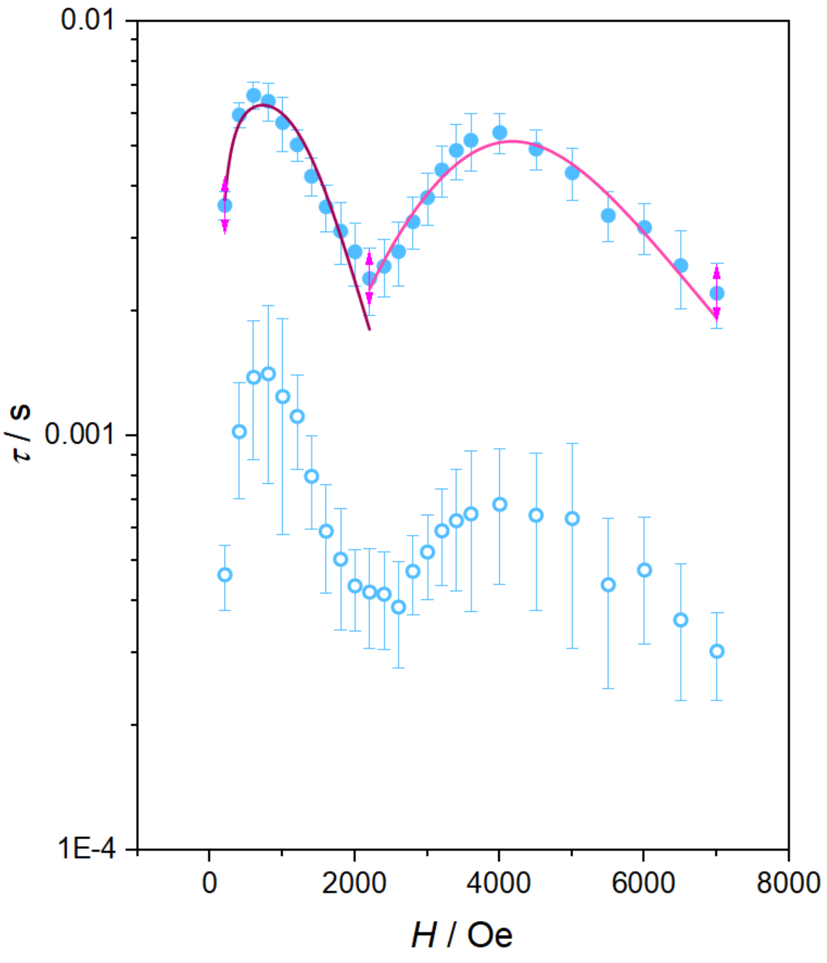

Figure S13. Magnetic field (H_{DC}) dependence of τ for **Er2** at 3.0 K described by two processes – longer τ_1 (full circles) and shorter τ_2 (open circles). Longer τ_1 was fitted with Equation 1 (main text) in two separate ranges. Details about the fits are collected in Table S8.

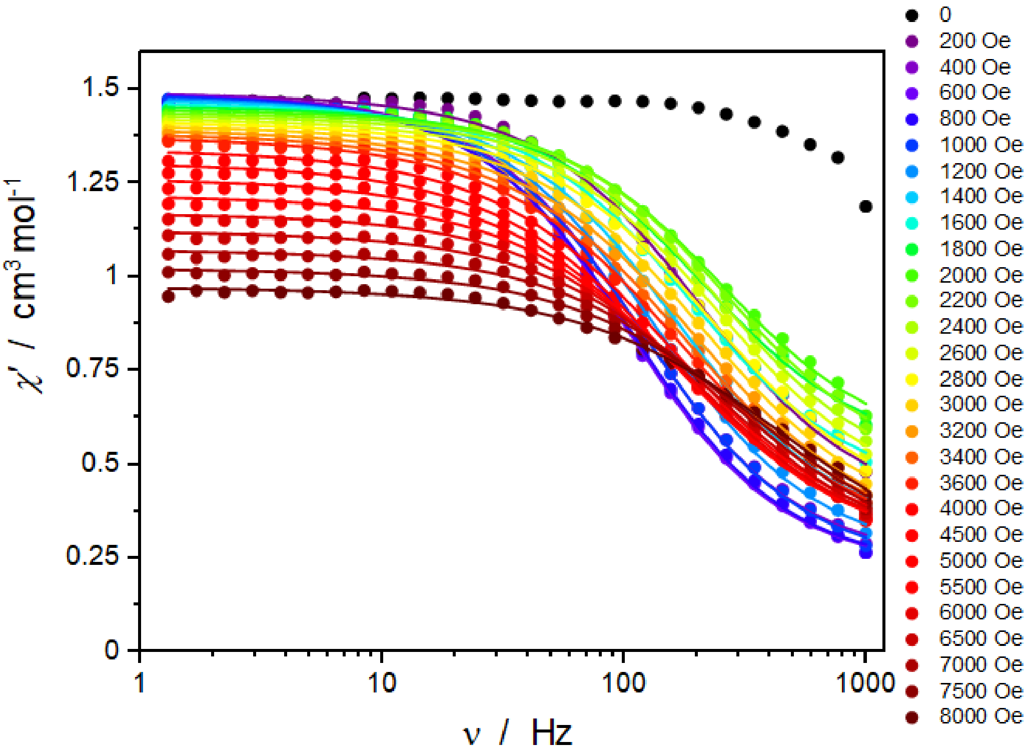

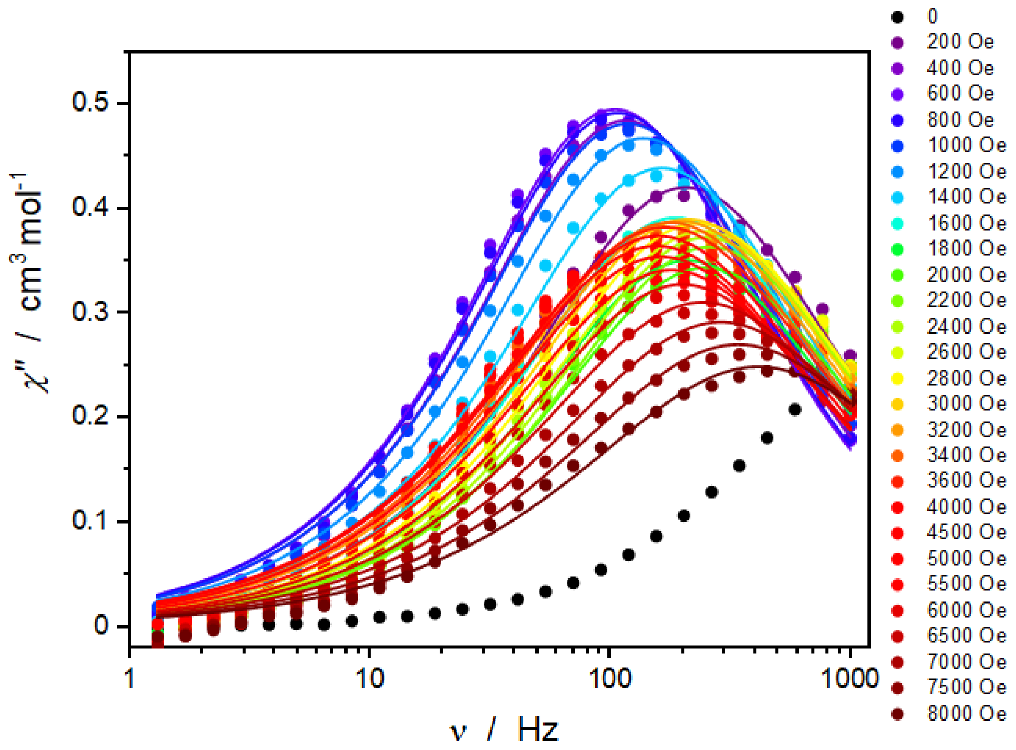

Figure S14. In-phase (χ') and out-of-phase (χ'') AC susceptibilities for **Er₂** at 4.5 K measured in various fields H_{DC} (0-8000 Oe). Values of α and τ parameters are presented in Table S6.

Table S6. Values of α and τ from fitting modified Debye function to τ(ν) dependencies for $\mathbf{Er_2}$ at 4.5 K.

***H* / Oe**	**α**	**τ / s**
200	0.19(1)	0.00077(2)
400	0.167(8)	0.00137(2)
600	0.161(8)	0.00152(2)
800	0.164(8)	0.00148(2)
1000	0.173(8)	0.00134(2)
1200	0.177(8)	0.00116(2)
1400	0.186(9)	0.00097(2)
1600	0.19(1)	0.00085(2)
1800	0.19(1)	0.00076(2)
2000	0.19(1)	0.00064(2)
2200	0.18(1)	0.00061(1)
2400	0.18(1)	0.00063(2)
2600	0.19(1)	0.00065(2)
2800	0.20(1)	0.00069(2)
3000	0.21(1)	0.00076(2)
3200	0.22(1)	0.00082(2)
3400	0.22(1)	0.00089(2)
3600	0.23(1)	0.00093(3)
4000	0.22(1)	0.00100(3)
4500	0.22(1)	0.00102(3)
5000	0.22(1)	0.00097(3)
5500	0.21(1)	0.00089(3)
6000	0.21(1)	0.00079(2)
6500	0.22(1)	0.00065(2)
7000	0.23(1)	0.00055(2)
7500	0.24(1)	0.00047(2)
8000	0.25(1)	0.00038(2)

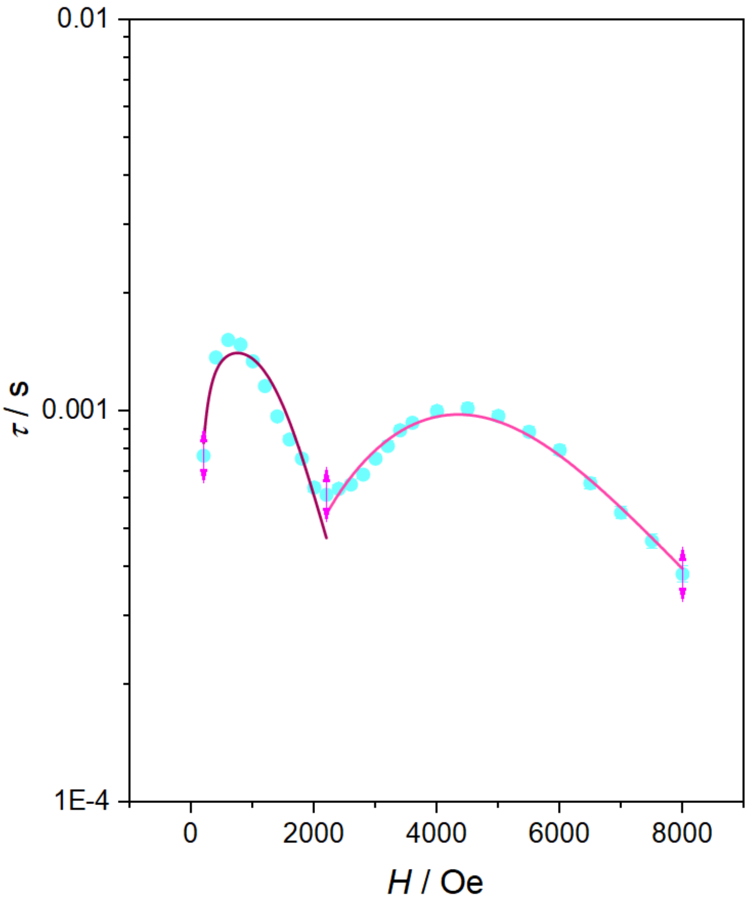

Figure S15. Magnetic field (H_{DC}) dependence of τ for **Er_2** at 4.5 K described by two processes – longer τ_1 (full circles) and shorter τ_2 (open circles). Longer τ_1 was fitted with Equation 1 (main text) in two separate ranges. Details about the fits are collected in Table S8.

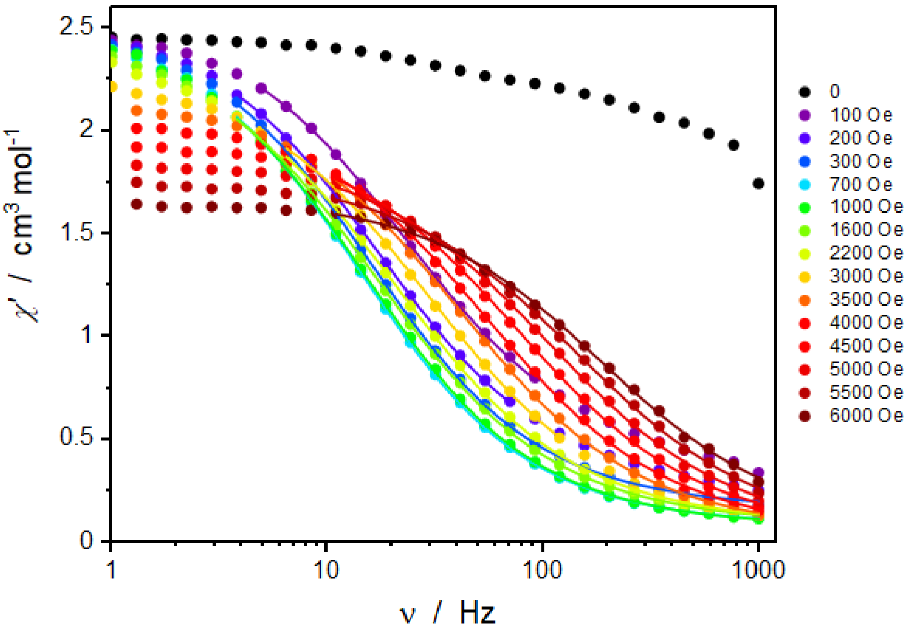

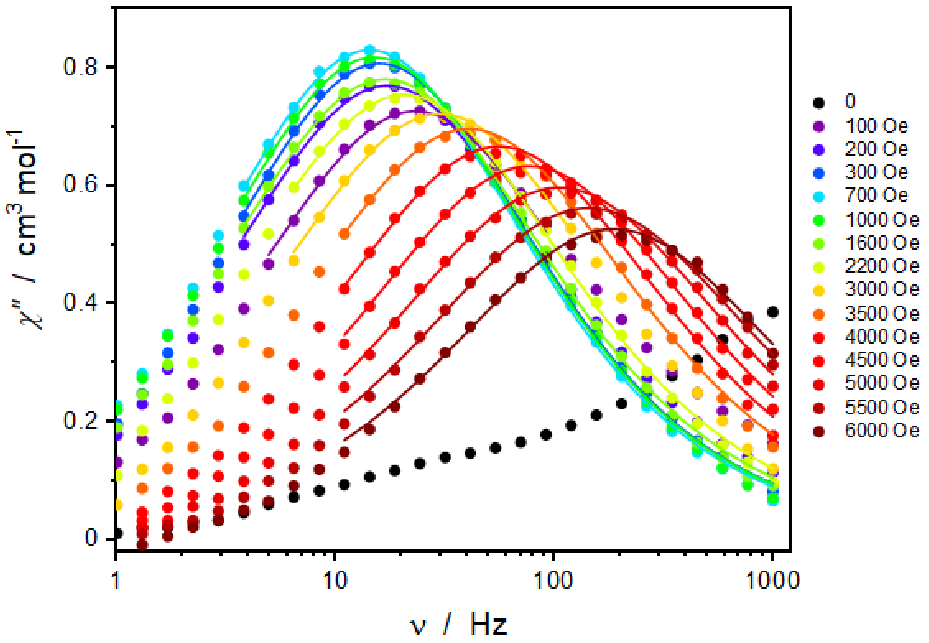

Figure S16. In-phase (χ') and out-of-phase (χ'') AC susceptibilities for **ErY@Y$_2$** at 4.0 K measured in various fields H_{DC} (0-6000 Oe). Only data for selected H_{DC} field values are shown on the graphs for better clarity. Values of α and τ parameters are presented in Table S7.

Table S7. Values of α and τ from fitting modified Debye function to τ(ν) dependencies for **ErY@Y_2** at 4.0 K.

H **/ Oe**	**α**	**τ / s**	**fitted range / Hz**
100	0.267(5)	0.00690(6)	5 - 92
200	0.257(4)	0.00929(6)	4 - 70
300	0.249(7)	0.0101(2)	4 - 1000
400	0.245(2)	0.01076(6)	4 - 1000
500	0.246(3)	0.01111(9)	4 - 1000
600	0.243(3)	0.01111(9)	4 - 1000
700	0.248(3)	0.0112(1)	4 - 1000
800	0.247(3)	0.0110(1)	4 - 1000
900	0.253(4)	0.0108(1)	4 - 1000
1000	0.253(3)	0.0106(1)	4 - 1000
1200	0.258(4)	0.0104(1)	4 - 1000
1400	0.263(3)	0.00992(8)	4 - 1000
1600	0.268(3)	0.00945(8)	4 - 1000
1800	0.270(3)	0.00891(7)	4 - 1000
2000	0.273(4)	0.00833(8)	4 - 1000
2200	0.274(4)	0.00772(8)	6 - 1000
2400	0.269(4)	0.00706(7)	6 - 1000
2600	0.272(4)	0.00646(7)	6 - 1000
2800	0.269(4)	0.00584(6)	6 - 1000
3000	0.278(5)	0.00532(5)	6 - 120
3500	0.276(5)	0.00392(5)	11 - 1000
4000	0.276(6)	0.00286(4)	11 - 1000
4500	0.276(6)	0.00207(2)	11 - 1000
5000	0.283(7)	0.00151(2)	11 - 1000
5500	0.282(7)	0.00111(2)	11 - 1000
6000	0.274(7)	0.00085(1)	11 - 1000

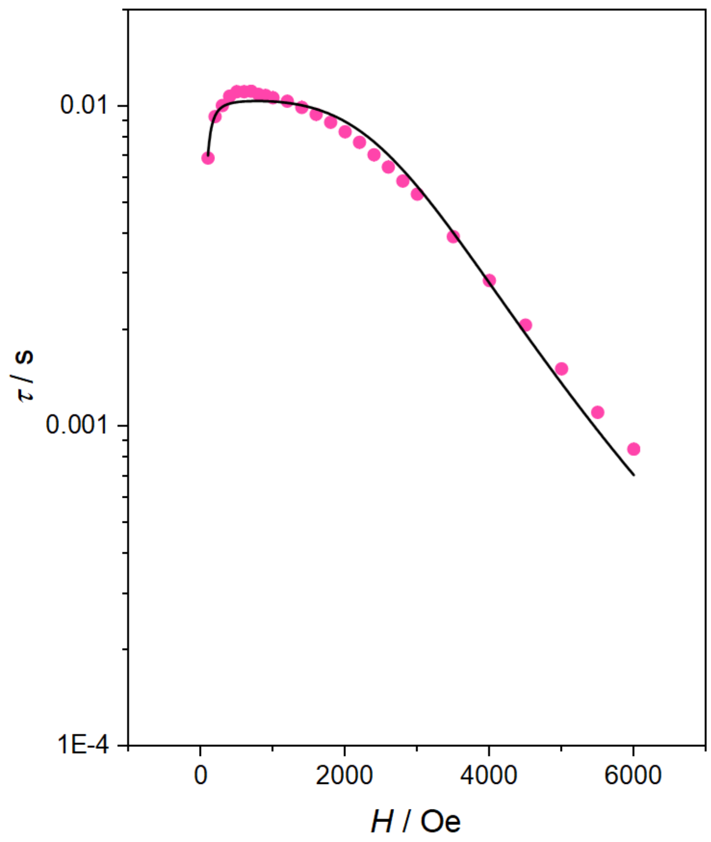

Figure S17. Magnetic field (H_{DC}) dependence of τ for **ErY@Y₂** at 4.0 K fitted with Equation 1 (main text). Details about the fits are collected in Table S8 below.

Table S8. Values of B_1, B_2, B_3 and R^2 for fitting $\tau(H)$ plot for **Er_2** at different temperatures (1.8 K, 3.0 K and 4.5 K) and for **ErY@Y_2** at 4.0 K.

	H_{DC} / Oe	T / K	B_1 / Oe^2s^{-1}	B_2 / $Oe^{-4}s^{-1}$	B_3 / s^{-1}	R^2
**Er_2**	200-2200	1.8	$2.95(47)\cdot10^6$	$1.41(18)\cdot10^{-11}$	104(4)	0.96081
		3.0	$4.99(84)\cdot10^6$	$1.74(24)\cdot10^{-11}$	145(8)	0.93576
		4.5	$2.22(63)\cdot10^7$	$6.20(95)\cdot10^{-11}$	651(40)	0.91121
	2200-7000 or 2200-8000	1.8	$1.25(12)\cdot10^9$	$1.64(16)\cdot10^{-13}$	42(14)	0.94398
		3.0	$2.02(17)\cdot10^9$	$1.91(17)\cdot10^{-13}$	21(18)	0.94783
		4.5	$6.30(46)\cdot10^9$	$4.66(34)\cdot10^{-13}$	520(54)	0.95577
**ErY@Y_2**	100-6000	4.0	$4.72(88)\cdot10^5$	$1.02(5)\cdot10^{-12}$	95(2)	0.99300

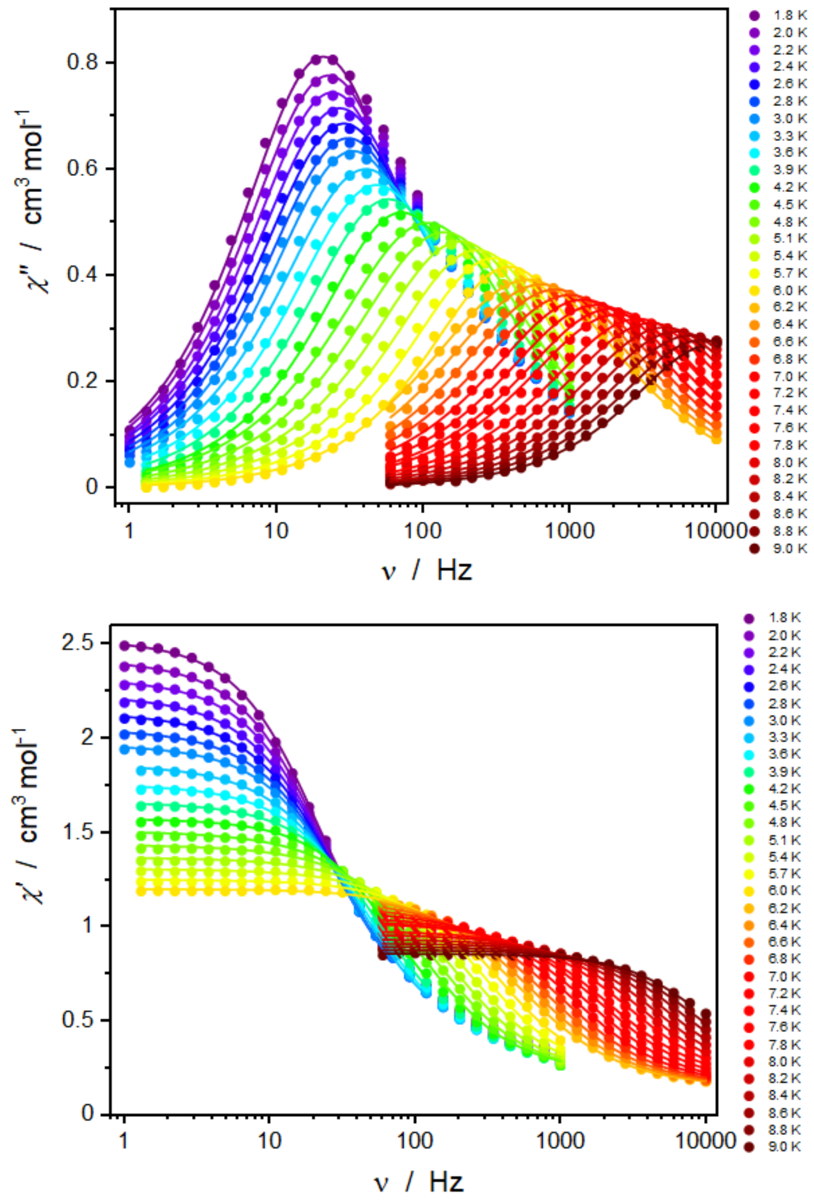

Figure S18. In-phase (χ') and out-of-phase (χ'') AC susceptibilities for **Er₂** at 700 Oe measured in various temperatures (1.8-9.0 K). Values of α and τ parameters are presented in Table S9.

Table S9. Values of α and τ from fitting modified Debye function to τ(ν) dependencies for **Er2** at 700 Oe.

MPMS			PPMS		
T **/ K**	**α**	**τ / s**	***T*** **/ K**	**α**	**τ / s**
1.8	0.110(8)	0.0076(2)	6.2	0.199(4)	$3.09(2)\cdot10^{-4}$
2.0	0.123(8)	0.0070(1)	6.4	0.194(4)	$2.49(2)\cdot10^{-4}$
2.2	0.119(8)	0.0066(1)	6.6	0.187(5)	$2.00(2)\cdot10^{-4}$
2.4	0.139(9)	0.0059(1)	6.8	0.184(6)	$1.61(2)\cdot10^{-4}$
2.6	0.136(9)	0.0055(1)	7.0	0.177(7)	$1.30(2)\cdot10^{-4}$
2.8	0.131(9)	0.0052(1)	7.2	0.177(6)	$1.04(1)\cdot10^{-4}$
3.0	0.124(9)	0.00478(9)	7.4	0.171(7)	$8.4(1)\cdot10^{-5}$
3.3	0.14(1)	0.00390(8)	7.6	0.166(7)	$6.79(9)\cdot10^{-5}$
3.6	0.134(9)	0.00331(7)	7.8	0.158(7)	$5.46(8)\cdot10^{-5}$
3.9	0.12(1)	0.00274(6)	8.0	0.153(8)	$4.45(8)\cdot10^{-5}$
4.2	0.11(1)	0.00223(5)	8.2	0.140(7)	$3.61(6)\cdot10^{-5}$
4.5	0.16392	0.00148(2)	8.4	0.125(8)	$2.97(6)\cdot10^{-5}$
4.8	0.15443	0.00115(2)	8.6	0.117(7)	$2.44(5)\cdot10^{-5}$
5.1	0.14372	$8.86(13)\cdot10^{-4}$	8.8	0.109(7)	$2.02(4)\cdot10^{-5}$
5.4	0.13235	$6.83(10)\cdot10^{-4}$	9.0	0.087(9)	$1.77(6)\cdot10^{-5}$
5.7	0.11568	$5.29(8)\cdot10^{-4}$			
6.0	0.09919	$4.12(6)\cdot10^{-4}$			

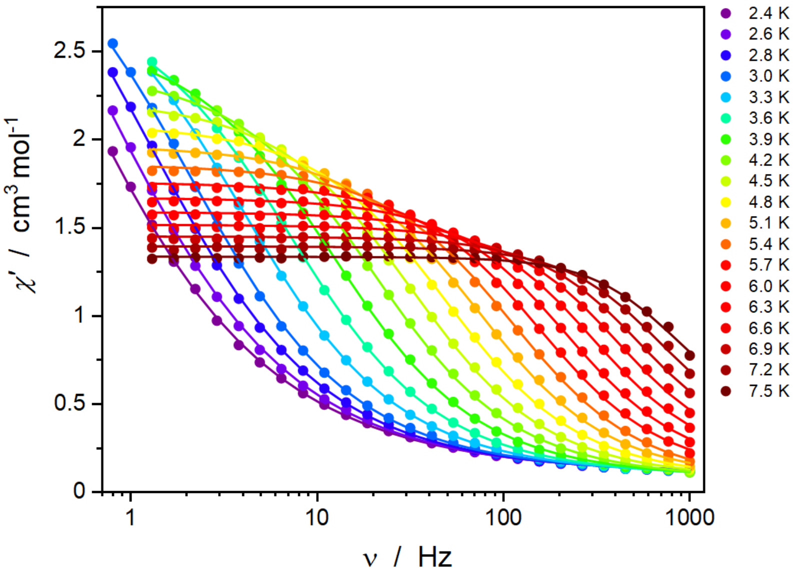

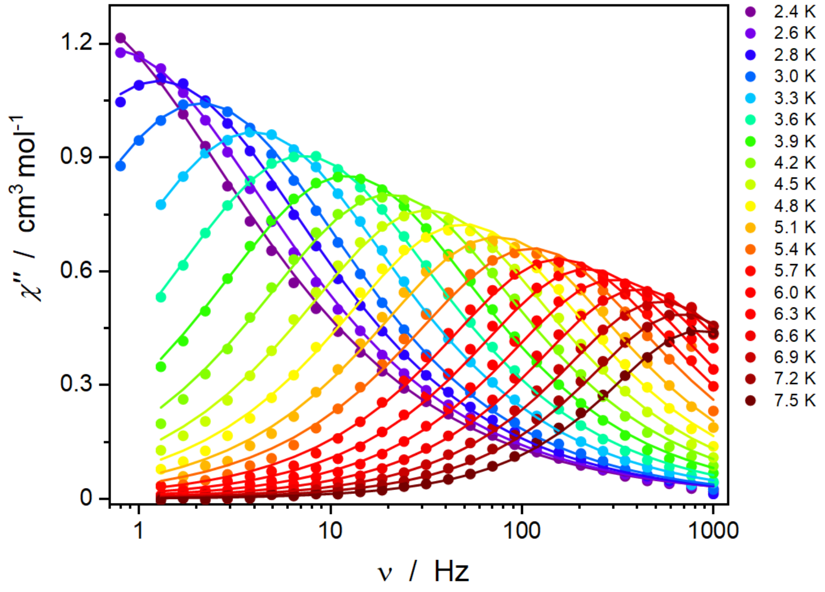

Figure S19. In-phase (χ') and out-of-phase (χ'') AC susceptibilities for **ErY@Y₂** at 700 Oe measured in various temperatures (1.8-7.5 K). Values of α and τ parameters are presented in Table S10.

Table S10. Values of α and τ from fitting modified Debye function to τ(ν) dependencies for **ErY@Y2** at 700 Oe.

***T* / K**	**α**	**τ / s**
2.4	0.386(4)	0.40(2)
2.6	0.352(4)	0.222(6)
2.8	0.319(3)	0.125(2)
3.0	0.291(3)	0.0757(8)
3.3	0.274(2)	0.0403(3)
3.6	0.255(2)	0.0222(1)
3.9	0.244(3)	0.01310(9)
4.2	0.232(4)	0.00788(7)
4.5	0.218(4)	0.00497(5)
4.8	0.208(5)	0.00324(3)
5.1	0.198(6)	0.00216(3)
5.4	0.196(9)	0.00142(3)
5.7	0.174(7)	0.00102(1)
6.0	0.156(7)	0.00073(1)
6.3	0.138(7)	0.000527(8)
6.6	0.116(7)	0.000389(7)
6.9	0.090(7)	0.000299(5)
7.2	0.065(9)	0.000233(5)
7.5	0.034(9)	0.000188(5)

Table S11. Fitting parameters from the analysis of ln$\tau(T^{-1})$ at H_{DC} = 700 Oe for **Er2** and **ErY@Y2**.

	**Er2**	**ErY@Y2**
H_{DC} / Oe	700	700
Frequency range / Hz	1-10000	1-1000
Temperature range / K	1.8-9.0	2.4-7.5
C_1 / $s^{-1}K^{-1}$	72(2)	-
C_2 / $s^{-1}K^{-n}$	$2.10(48)\cdot10^{-3}$	$8.43(30)\cdot10^{-3}$
n	7.75(12)	6.69(2)
R^2	0.99835	0.99979

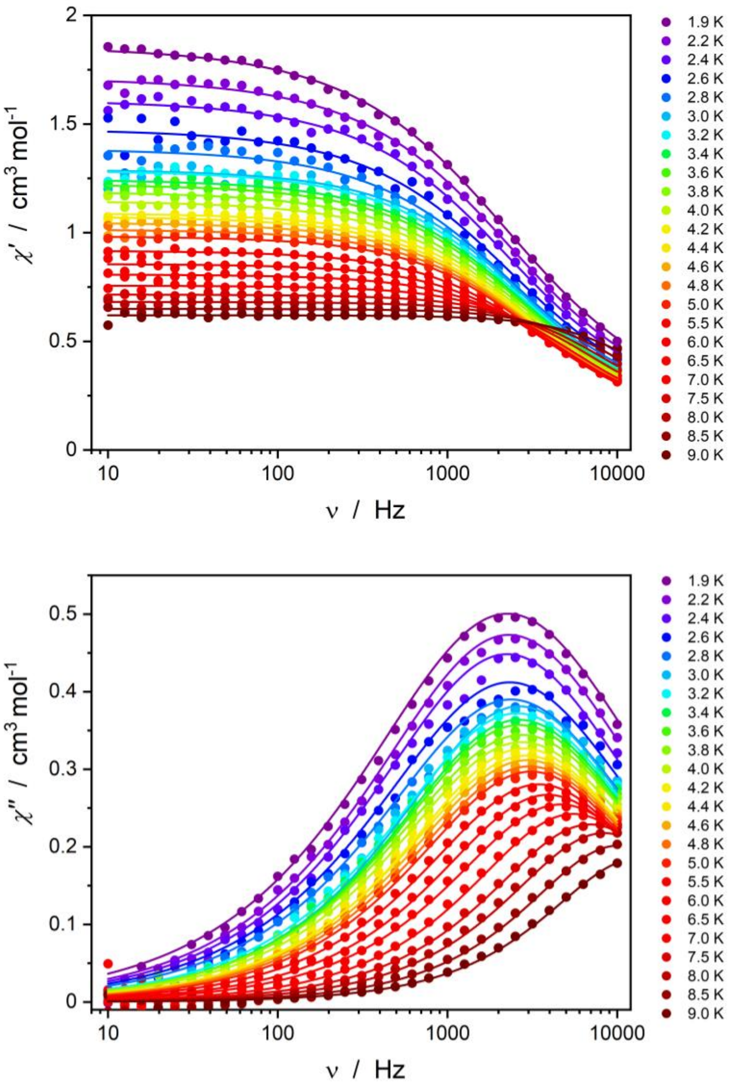

Figure S20. In-phase (χ') and out-of-phase (χ'') AC susceptibilities for **Er_2** without external magnetic field measured in various temperatures (1.9-9.0 K). Values of α and τ parameters are presented in Table S12.

Table S12. Values of α and τ from fitting modified Debye function to τ(ν) dependencies for **Er_2** at $H_{DC} = 0$.

***T* / K**	**α**	**τ / s**
1.9	0.321(6)	$7.05(14)\cdot10^{-5}$
2.2	0.299(8)	$6.92(19)\cdot10^{-5}$
2.4	0.287(11)	$7.08(24)\cdot10^{-5}$
2.6	0.285(18)	$6.85(38)\cdot10^{-5}$
2.8	0.276(17)	$6.72(35)\cdot10^{-5}$
3.0	0.241(16)	$6.01(27)\cdot10^{-5}$
3.2	0.258(11)	$6.20(21)\cdot10^{-5}$
3.4	0.250(9)	$6.02(16)\cdot10^{-5}$
3.6	0.251(10)	$6.00(18)\cdot10^{-5}$
3.8	0.263(7)	$5.79(12)\cdot10^{-5}$
4.0	0.248(9)	$5.81(16)\cdot10^{-5}$
4.2	0.222(11)	$5.63(17)\cdot10^{-5}$
4.4	0.238(10)	$5.50(16)\cdot10^{-5}$
4.6	0.230(11)	$5.39(17)\cdot10^{-5}$
4.8	0.225(11)	$5.23(16)\cdot10^{-5}$
5.0	0.214(15)	$5.11(21)\cdot10^{-5}$
5.5	0.205(9)	$4.59(12)\cdot10^{-5}$
6.0	0.185(17)	$4.07(19)\cdot10^{-5}$
6.5	0.180(11)	$3.43(11)\cdot10^{-5}$
7.0	0.145(17)	$2.89(14)\cdot10^{-5}$
7.5	0.131(14)	$2.30(10)\cdot10^{-5}$
8.0	0.113(13)	$1.90(9)\cdot10^{-5}$
8.5	0.0834(19)	$1.57(10)\cdot10^{-5}$
9.0	0.0759(36)	$1.20(19)\cdot10^{-5}$

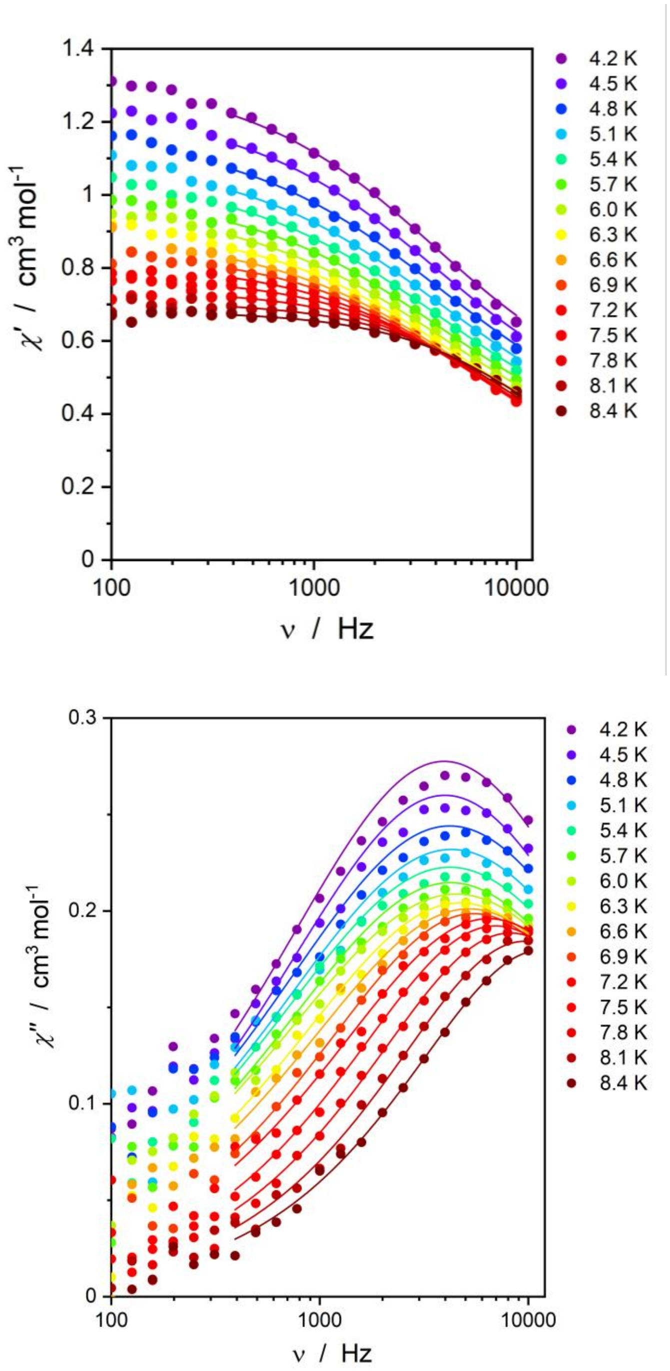

Figure S21. In-phase (χ') and out-of-phase (χ'') AC susceptibilities for **ErY@Y$_2$** without external magnetic field measured in various temperatures (4.1-8.4 K). Values of α and τ parameters are presented in Table S13.

Table S13. Values of α and τ from fitting modified Debye function to τ(ν) dependencies for **ErY@Y2** at $H_{DC} = 0$.

***T* / K**	**α**	**τ / s**
4.2	0.321(16)	$4.04(16)\cdot10^{-5}$
4.5	0.322(14)	$4.00(14)\cdot10^{-5}$
4.8	0.349(15)	$3.79(16)\cdot10^{-5}$
5.1	0.352(16)	$3.72(16)\cdot10^{-5}$
5.4	0.354(11)	$3.79(11)\cdot10^{-5}$
5.7	0.343(12)	$3.76(12)\cdot10^{-5}$
6.0	0.358(12)	$3.53(12)\cdot10^{-5}$
6.3	0.327(8)	$3.38(8)\cdot10^{-5}$
6.6	0.326(11)	$3.07(10)\cdot10^{-5}$
6.9	0.287(8)	$2.91(7)\cdot10^{-5}$
7.2	0.291(13)	$2.46(10)\cdot10^{-5}$
7.5	0.248(9)	$2.28(7)\cdot10^{-5}$
7.8	0.227(9)	$1.94(6)\cdot10^{-5}$
8.1	0.203(12)	$1.66(7)\cdot10^{-5}$
8.4	0.200(14)	$1.36(8)\cdot10^{-5}$

Table S14. Fitting parameters from the analysis of lnτ(T^{-1}) (H_{DC} = 0) for **Er$_2$** and **ErY@Y$_2$**.

	**Er$_2$**	**ErY@Y$_2$**
H_{DC} / Oe	0	0
Frequency range / Hz	1-10000	1-1000
Temperature range / K	1.9-9.0	4.2-8.4
C_0 / s^{-1}	11572(350)	25044(300)
C_1 / s^{-1}K^{-1}	1335(126)	0 (fixed)
C_2 / s^{-1}K^{-n}	0.097(71)	0.0016(12)
n	6.08(34)	8.08(35)
R^2	0.99652	0.99615

COMPUTATIONAL DETAILS

The *ab initio* calculations for **Er₂**, were performed using the crystal structure taken from the single-crystal X-ray diffraction (scXRD) experiment without geometry optimization. The molecular cluster that was used in the computational procedure is visualized in Figure S22 together with calculated main magnetic axes scaled with the corresponding g-tensor components, and consisted of a whole two-center molecule with one Er^{III} ion substituted by the closed-shell, diamagnetic Y^{III} ion to enable multiconfigurational treatment of the fragment. In order to examine local magnetic properties of Er^{III} centers, State Average Complete Active Space Self-Consistent Field (SA-CASSCF) calculations were performed using the OpenMolcas software.[3] Scalar relativistic effects were taken into account by employing two-component second-order Douglas-Kroll-Hess (DKH2) Hamiltonian together with relativistic Atomic Natural Orbital basis sets of the ANO-RCC type.[4-6] To save disk space for computations of this huge cluster (216 atoms), the Cholesky decomposition of ERI-s (electron repulsion integrals) was used with the $1.0{\cdot}10^{-8}$ threshold. The used model employed the VTZP basis function quality for Er^{III} centers, the VDZP for oxygen atoms occupying the first coordination sphere, and the VDZ for the others. A description of the employed basis set and its contractions are presented in Table S15. In the CASSCF step, the active space was composed of seven 4f-orbitals of Er^{III} centers with 11 active electrons – CAS(11in7), and 35 quartets, and 112 doublets states arising from different possible electron distributions within $4f^{11}$ configuration were evaluated. In the next step, all of optimized as spin-free states in the CASSCF step were mixed within the Restricted Active Space State Interaction (RASSI) submodule by Spin-Orbit-Coupling (SOC) within the atomic mean-field (AMFI) approximation.[7, 8] In the final step, the resulting 364 spin-orbit states were analyzed using the SINGLE_ANISO module to obtain main magnetic axes and the pseudo-*g*-tensors of each Kramers doublet, simulate $\chi_M(T)$ and $M(H)$ magnetic dependences (Figures 2 and 3), and decompose the ground SO states into ones with a definite projection of the total momentum on the located quantization *z*-axis of the ground Kramers doublet.[9, 10] The energy splitting of the ground $^4I_{15/2}$ multiplet of Er^{III} center together with pseudo-*g*-tensor components and the composition of the ground doublet in the $|J = 15/2, m_J>$ basis are presented in Table S16.

After obtaining the local *ab initio* magnetic properties of Er^{III} ions, two centers are related by the spatial inversion, we employed the POLY_ANISO module to simulate the magnetic interaction

of two adjacent centers in each bimetallic **Er2** molecule. We took into account both dipole-dipole and exchange interactions. The former are treated exactly using the positions and magnetic momenta of Er^{III} centers found during the *ab initio* procedure:

$$H_{dip} = \frac{\mu_0}{4\pi r^3}\left[\boldsymbol{\mu_1} \cdot \boldsymbol{\mu_2} - \frac{3}{r^2}(\boldsymbol{\mu_1} \cdot \boldsymbol{r})(\boldsymbol{\mu_2} \cdot \boldsymbol{r})\right] \quad \text{(Eq. S1)}$$

where $\boldsymbol{\mu_1}$ and $\boldsymbol{\mu_2}$ are magnetic dipole moment operators for two Er^{III} centers and $\boldsymbol{r}$ is the distance between them. The exchange interaction is accounted for within the Lines model[11] utilizing a single, effective isotropic exchange parameter $\boldsymbol{J_{Lines}}$ with the Hamiltonian equation:

$$H_{exch} = -\boldsymbol{J_{Lines}} \cdot \tilde{\boldsymbol{S}}_{\boldsymbol{1}} \cdot \tilde{\boldsymbol{S}}_{\boldsymbol{2}} \quad \text{(Eq. S2)}$$

where $\tilde{\boldsymbol{S}}_{\boldsymbol{1}}$ and $\tilde{\boldsymbol{S}}_{\boldsymbol{2}}$ are previously found *ab initio* pseudo-spin operators ($\boldsymbol{S} = 15/2$) corresponding to the states with definite projections on the z-quantization axis of the ground Kramers doublet (the same used for the decomposition in Tables S16). Therefore, the exchange manifold has a dimension of 16 x 16 exchange states, and the rest of the excited states are treated as local states on each magnetic site, not participating in exchange but with their contributions accounted for the simulation of magnetic properties. In this regard, the magnitude of the obtained exchange parameter cannot be compared to the usual, phenomenological pure spin Hamiltonians widely present in the literature. The $\boldsymbol{J_{Lines}}$ effective exchange parameter is the only unknown in the model, and it is found by fitting the simulated $\chi_M(T)$ dependencies to the experimental data (Figure 3). We ended with $\boldsymbol{J_{Lines}} = \boldsymbol{-0.3\ cm^{-1}}$ and included also a small effective $zJ = -0.1$ cm^{-1} parameter to improve fitting quality by accounting for intermolecular interactions. After construction and diagonalization of the exchange Hamiltonian, we ended up with new exchange energy states, which were also analyzed for *g*-tensors and tunneling splitting between the Ising doublets. Note that, after the coupling, investigated systems are no longer Kramers ones; therefore, the degeneracy of doublets is lifted, and $g_{x,}$ g_y components of the pseudo-*g*-tensors are zero from the definition. The only relevant factor, then, is the tunneling splitting between them. The energies, tunnelling splitting, and g_z components of pseudo-*g*-tensors are reported in Table S17. Additionally, we simulated Zeeman splitting (*x*, *y*, *z* axes and averaged over 17 directions over hemisphere) for the two lowest pseudo-doublets Figure 8 to investigate the avoided level crossing with comparison to field dependence of relaxation time τ.

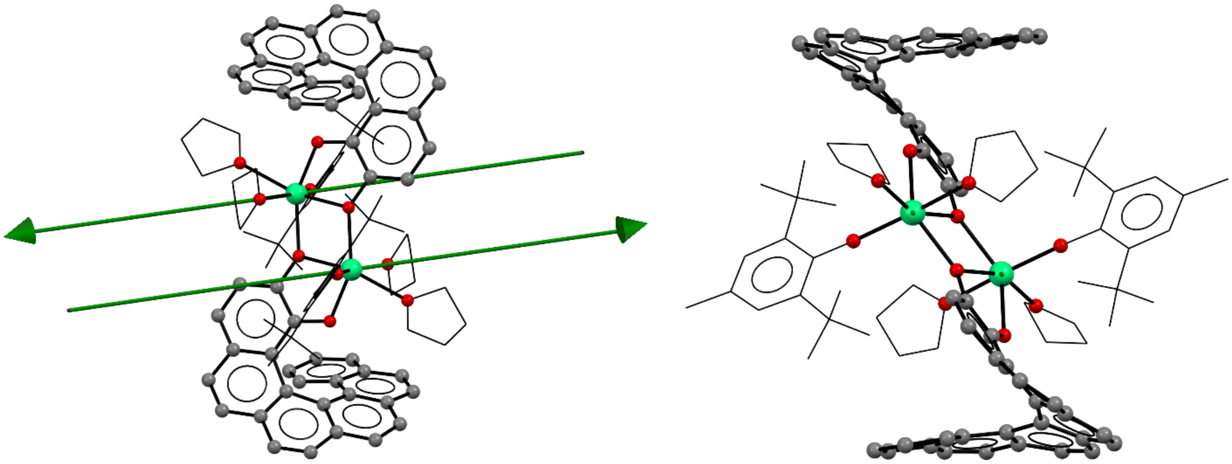

Figure S22. The molecular cluster that was used in the computational procedure together with calculated main magnetic axes (left – arbitrarily chosen orientation, right – view along magnetic axes). The magnetic axes were scaled with the corresponding g-tensor components, therefore only axes along z direction are visible.

Table S15. Description and contractions of the basis set employed in the *ab initio* calculations of the Er^{III} crystal field in **Er$_2$**.

**Basis set**
Er.ANO-RCC-VTZP 8S7P5D3F2G1H
Y.ANO-RCC-VDZ 6S5P3D
O.ANO-RCC-VDZP 3S2P1D (for the first coordination sphere)<br>O.ANO-RCC-VDZ 3S2P (for others)
C.ANO-RCC-VDZ 3S2P
H.ANO-RCC-VDZ 2S

Table S16. Summary of the energy splitting of the $^{4}I_{15/2}$ multiplet of Er^{III} centers in **Er$_2$** with the pseudo-*g*-tensors of each Kramers doublet and the composition in the $|m_J\rangle$ basis of the ground state.

<table>
<tr><th colspan="6">energy and pseudo-g-tensor components (g_x, g_y, g_z) of 8 ground Kramers doublets</th></tr>
<tr><th rowspan="2" colspan="3">energy / cm^{-1}</th><th colspan="3">pseudo-g-tensor components</th></tr>
<tr><th>g_x</th><th>g_y</th><th>g_z</th></tr>
<tr><td colspan="3">0.000</td><td>0.0504</td><td>0.1146</td><td>16.9257</td></tr>
<tr><td colspan="3">85.373</td><td>1.2254</td><td>2.3480</td><td>13.2349</td></tr>
<tr><td colspan="3">135.802</td><td>1.1521</td><td>3.4724</td><td>12.3470</td></tr>
<tr><td colspan="3">186.868</td><td>0.9164</td><td>3.5154</td><td>9.2208</td></tr>
<tr><td colspan="3">249.433</td><td>1.9848</td><td>4.7834</td><td>8.8348</td></tr>
<tr><td colspan="3">317.974</td><td>1.4394</td><td>2.4114</td><td>13.8659</td></tr>
<tr><td colspan="3">368.248</td><td>0.0671</td><td>1.1751</td><td>13.9761</td></tr>
<tr><td colspan="3">419.836</td><td>0.5776</td><td>1.3121</td><td>16.2585</td></tr>
<tr><th colspan="6">composition of the ground Kramers doublets in the $|m_J\rangle$ the basis on the quantization axes within J = 15/2 manifold</th></tr>
<tr><th>1st doublet</th><th>2nd doublet</th><th>3rd doublet</th><th>4th doublet</th><th>5th doublet</th><th>6th doublet</th></tr>
<tr><td>82.1% |±15/2></td><td>0.1% |±15/2></td><td>0.0% |±15/2></td><td>2.1% |±15/2></td><td>1.5% |±15/2></td><td>12.4% |±15/2></td></tr>
<tr><td>2.2% |±13/2></td><td>59.4% |±13/2></td><td>5.3% |±13/2></td><td>3.2% |±13/2></td><td>8.3% |±13/2></td><td>16.0% |±13/2></td></tr>
<tr><td>10.7% |±11/2></td><td>15.7% |±11/2></td><td>5.6% |±11/2></td><td>23.0% |±11/2></td><td>7.6% |±11/2></td><td>27.7% |±11/2></td></tr>
<tr><td>3.4% |±9/2></td><td>4.9% |±9/2></td><td>14.1% |±9/2></td><td>26.3% |±9/2></td><td>12.7% |±9/2></td><td>23.1% |±9/2></td></tr>
<tr><td>1.1% |±7/2></td><td>13.4% |±7/2></td><td>19.1% |±7/2></td><td>4.1% |±7/2></td><td>13.0% |±7/2></td><td>7.9% |±7/2></td></tr>
<tr><td>0.1% |±5/2></td><td>3.3% |±5/2></td><td>39.0% |±5/2></td><td>2.5% |±5/2></td><td>2.7% |±5/2></td><td>2.7% |±5/2></td></tr>
<tr><td>0.1% |±3/2></td><td>2.0% |±3/2></td><td>15.0% |±3/2></td><td>24.8% |±3/2></td><td>9.0% |±3/2></td><td>8.3% |±3/2></td></tr>
<tr><td>0.2% |±1/2></td><td>1.3% |±1/2></td><td>1.8% |±1/2></td><td>14.1% |±1/2></td><td>45.1% |±1/2></td><td>2.9% |±1/2></td></tr>
</table>

Table S17. Summary of the energy splitting of the first 60 exchange states in **Er_2** with the pseudo-*g*-tensor g_z components and tunneling splitting between the Ising doublets.

energy, g_z components of pseudo-*g*-tensors, and tunneling splitting between the Ising doublets			
energy / cm^{-1}		g_z	tunneling splitting / cm^{-1}
0	0	6.49E-05	3.67E-09
1.179	1.179	4.33E-05	33.84854
85.476	85.476	0.000181	4.06E-09
85.559	85.56	0.000753	6.99E-09
86.304	86.305	0.000699	29.88815
86.507	86.507	0.000494	29.87988
136.203	136.205	0.002231	16.01447
136.219	136.223	0.004083	16.01915
136.562	136.564	0.00264	1.92E-07
136.576	136.579	0.002602	1.94E-07
170.962	170.991	0.02889	1.06E-08
171.683	171.7	0.016926	26.43304
187.215	187.218	0.002697	6.43E-09
187.258	187.259	0.000918	5.82E-09
187.654	187.658	0.004242	24.00149
187.7	187.701	0.001397	23.99366
221.582	221.593	0.011466	2.05E-08
221.677	221.735	0.058563	5.88272
221.773	221.831	0.057944	8.05E-08
221.955	221.965	0.010039	17.90289
249.853	249.853	0.00017	7.17E-09
249.869	249.87	0.000917	7.53E-09

250.173	250.175	0.001927	21.64047
250.192	250.194	0.002029	21.64329
271.844	271.894	0.049224	2.34E-09
272.465	272.476	0.010746	22.69761
272.609	272.647	0.037682	1.71E-08
272.68	272.708	0.028654	1.77E-08
272.931	272.948	0.017	20.60348
273.061	273.159	0.097586	19.9143

REFERENCES

(1) Llunell, M.; Casanova, D.; Cirera, J.; Alemany, P.; Alvarez, S. SHAPE, version 2.1. *Barcelona, Spain* **2013**.

(2) Steiner, T. The Hydrogen Bond in the Solid State. *Angew. Chem. Int. Ed.* **2002**, *41* (1), 48-76. DOI: 10.1002/1521-3773(20020104)41:1<48::AID-ANIE48>3.0.CO;2-U.

(3) Fdez. Galván, I.; Vacher, M.; Alavi, A.; Angeli, C.; Aquilante, F.; Autschbach, J.; Bao, J. J.; Bokarev, S. I.; Bogdanov, N. A.; Carlson, R. K.; et al. OpenMolcas: From Source Code to Insight. *Journal of Chemical Theory and Computation* **2019**, *15* (11), 5925-5964. DOI: 10.1021/acs.jctc.9b00532.

(4) Roos, B. O.; Lindh, R.; Malmqvist, P.-Å.; Veryazov, V.; Widmark, P.-O. Main Group Atoms and Dimers Studied with a New Relativistic ANO Basis Set. *J. Phys. Chem. A* **2004**, *108* (15), 2851-2858. DOI: 10.1021/jp031064+.

(5) Roos, B. O.; Lindh, R.; Malmqvist, P.-Å.; Veryazov, V.; Widmark, P.-O. New Relativistic ANO Basis Sets for Transition Metal Atoms. *J. Phys. Chem. A* **2005**, *109* (29), 6575-6579. DOI: 10.1021/jp0581126.

(6) Roos, B. O.; Lindh, R.; Malmqvist, P.-Å.; Veryazov, V.; Widmark, P.-O.; Borin, A. C. New Relativistic Atomic Natural Orbital Basis Sets for Lanthanide Atoms with Applications to the Ce Diatom and LuF_3. *J. Phys. Chem. A* **2008**, *112* (45), 11431-11435. DOI: 10.1021/jp803213j.

(7) Malmqvist, P. Å.; Roos, B. O.; Schimmelpfennig, B. The restricted active space (RAS) state interaction approach with spin–orbit coupling. *Chemical Physics Letters* **2002**, *357* (3), 230-240. DOI: 10.1016/S0009-2614(02)00498-0.

(8) Heß, B. A.; Marian, C. M.; Wahlgren, U.; Gropen, O. A mean-field spin-orbit method applicable to correlated wavefunctions. *Chemical Physics Letters* **1996**, *251* (5), 365-371. DOI: 10.1016/0009-2614(96)00119-4.

(9) Chibotaru, L. F.; Ungur, L. Ab initio calculation of anisotropic magnetic properties of complexes. I. Unique definition of pseudospin Hamiltonians and their derivation. *The Journal of Chemical Physics* **2012**, *137* (6). DOI: 10.1063/1.4739763 (acccessed 6/10/2024).

(10) Ungur, L.; Chibotaru, L. F. Ab Initio Crystal Field for Lanthanides. *Chem. Eur. J.* **2017**, *23* (15), 3708-3718. DOI: 10.1002/chem.201605102.

(11) Lines, M. E. Orbital Angular Momentum in the Theory of Paramagnetic Clusters. *The Journal of Chemical Physics* **1971**, *55* (6), 2977-2984. DOI: 10.1063/1.1676524.
